# “It’s just part of who I am…” Living with chronic headache: voices from the CHESS trial, a qualitative study

**DOI:** 10.1186/s12883-024-03779-w

**Published:** 2024-08-02

**Authors:** Vivien P. Nichols, David R. Ellard, Frances E. Griffiths, Martin Underwood, Kirstie L. Haywood, Stephanie J. C. Taylor, On behalf of the CHESS team (Consortium), Vivien Nichols, Frances Griffiths, Felix Achana, Dawn Carnes, Sandra Eldridge, Siew Wan Hee, Helen Higgins, Dipesh Mistry, Hema Mistry, Sian Newton, Chloe Norman, Emma Padfield, Shilpa Patel, Stavros Petrou, Tamar Pincus, Rachel Potter, Harbinder Sandhu, Kimberley Stewart, Manjit Matharu

**Affiliations:** 1 Warwick Clinical Trials Unit, Warwick Medical School, University of Warwick, Coventry, CV4 7AL UK; 2 University Hospitals Coventry and Warwickshire, Clifford Bridge Road, Coventry, CV2 2DX UK; 3 Division of Health Sciences, Warwick Medical School, University of Warwick, Coventry, CV4 7AL UK; 4 Warwick Medical School, Warwick Research in Nursing, University of Warwick, Coventry, CV4 7AL UK; 5 Wolfson Institute for Population Health, Barts and The London School of Medicine and Dentistry, Queen Mary University of London, London, E1 2AB UK

**Keywords:** Lived Experiences, Chronic Headache, Qualitative, Pen Portraits

## Abstract

**Background:**

Between 2015 and 2019 the Chronic Headache Education and Self-management Study (CHESS) developed and tested a supportive self-management approach that aimed to improve outcomes for people with chronic migraine or chronic tension type headache with/without episodic migraine. However, a paucity of qualitative research which explored the lived experiences of people with chronic headache was evidenced. In response, we undertook to explore the experiences of living with chronic headaches of people who participated in the CHESS study.

**Methods:**

We adopted qualitative methodologies, inviting participants in the CHESS study to participate in semi-structured interviews. In phase 1 (feasibility study), a thematic analysis was conducted. In phase 2 (main CHESS trial), interviews were informed by topic guides developed from our learning from the phase 1 interviews. Pen portrait methodology and thematic analysis was employed allowing us to explore the data longitudinally.

**Results:**

Phase 1, 15 interviews (10 female) age range 29 to 69 years (median 47 years) revealed the complexities of living with chronic headache. Six overarching themes were identified including the emotional impact and the nature of their headaches. Phase 2, included 66 interviews (26 participants; median age group 50s (range 20s-60s); 20 females. 14 were interviewed at three points in time (baseline, 4 and 12 months) Through an iterative process four overlapping categories of headache impact emerged from the data and were agreed: i) ‘I will not let headaches rule my life’; ii) ‘Headaches rule my life’; iii) ‘Headaches out of control—something needs to change’; and iv) ‘Headaches controlled—not ruling my life’. One of these categories was assigned to each pen portrait at each timepoint.

The remaining 12 participants were interviewed at two time points during a year; pen portraits were again produced. Analysis revealed that the headache impact categories developed above held true in this sample also providing some validation of the categories.

**Conclusions:**

These data give an insight into the complexities of living with chronic headache. Chronic headache is unpredictable, permeating all aspects of an individual’s life; even when an individual feels that their headache is controlled and not interfering, this situation can rapidly change. It shows us that more work needs to be done both medically and societally to help people living with this often-hidden condition.

**Trial registration:**

ISRCTN79708100

**Supplementary Information:**

The online version contains supplementary material available at 10.1186/s12883-024-03779-w.

## Background

Migraines are often thought of as bad headaches which is a gross simplification of a condition which can be unpredictable and life changing [1]. Around 2–4% of people meet an epidemiological definition of chronic headache; that is, headaches on 15 or more days per month for at least three months [2]. The term chronic headache encompasses all chronic headache disorders, [3] including the primary headache disorders, which do not have secondary organic aetiology; chronic migraine and chronic tension type headache. It also encompasses medication overuse headache, a secondary headache disorder caused by using headache medication on either; ≥ 10 days per month if taking triptans (a migraine specific medication) or opioids, and ≥ 15 days a month if taking simple analgesia such as paracetamol or ibuprofen.

Between 2015 and 2019 we delivered the Chronic Headache Education and Self-management Study (CHESS) in the UK, funded by the UK National Institute of Health and Social Care Research (NIHR), Programme Grants for Applied Research. The main objective of CHESS was to test a supportive self-management approach to improve outcomes for people with chronic migraine or chronic tension type headache with/without episodic migraine. People with and without medication overuse headache were included. Recruitment was through NHS general medical practice [4]. Drawing on current best practice and the experiences of people with chronic headaches, a self-management support programme for people living with chronic headache was developed. This work is described in detail elsewhere [4–9].

As part of CHESS, we found a paucity of qualitative research looking specifically at the lived experiences of those who live with chronic headache. Our qualitative evidence synthesis (totalling *n* = 73 participants) identified only four papers meeting the inclusion criteria [10]. The synthesis of these papers showed three overarching themes: i) headache as a driver of behaviour (direct or indirect); ii) the spectre of headache (describing the emotional impact of this unpredictable condition); and iii) strained relationships (with friends, family and the medical profession) [10]. One of the recommendations from this review was that more research was needed to understand what it is like living with chronic headaches and how it affects people’s quality of life.

Whist the CHESS trial was large and adequately powered, no detectable effect was found for the intervention on the primary outcome (health-related quality of life) at 12-months [8, 11]. However, the CHESS programme of research included a considerable number of qualitative interviews. The interviews aimed to ensure that the voices of those living with chronic headaches informed both intervention development and contributed to the design, delivery, and interpretation of the study as a whole. This work is reported elsewhere [5]. During these interviews participants shared their stories of living with chronic headache. Here we present an analysis and synthesis of our participants’ experiences of living with chronic headaches.

## Methods

The study is presented in line with the Consolidated criteria for reporting qualitative research (COREQ) [12]. Data presented in this paper are from interviews conducted during the CHESS study. Two study phases are described: i) Phase one – feasibility study (April 2016 – January 2017); and ii) Phase two—main trial (RCT; April 2017 – March 2019). Participants living with chronic headache were defined as experiencing headache on 15 or more days per month for at least three months. In both phases, interview participants were recruited via English GP practices. All participants in phase 2 of this interview study were also CHESS trial participants [4]. All participants gave separate informed consent to participate in the trial and for the interviews. No formal diagnosis was carried out with participants as part of the interview studies. However, each participant was given a classification of their headache type as part of the CHESS study overall.

### Phase 1: Initial interviews

Overall, 131 people with headache disorders took part in the feasibility phase of the CHESS research programme. Participants who expressed an interest in taking part were purposively sampled to obtain a range of headache type, age, gender, and location. Participants in these initial interviews were not exposed to the CHESS interventions in any way. We used a semi structured schedule approach for face-to-face interviews exploring participants lived experience of chronic headache. Interview topic guides are provided as supplementary material. (Supplementary materials 1, pages 1–4).

### Phase 2: Main study interviews

Of 736 participants in the main CHESS trial, 396 (54%) had chronic migraine, 331 (45%) had chronic tension type headache and episodic migraine, and nine (1%) had chronic tension type headache only.

Participants in the main trial were approached if they had expressed an interest in participating in an interview as part of the main trial consent process. Those who consented were first interviewed post baseline assessment and pre-randomisation (when they had not yet been informed as to which arm of the study they were allocated); they were also invited for follow up interviews at four and 12-months. We found however that because we recruited participants before they were randomised that the sample of those who were taking part was giving more participants from the control arm of the trial. Therefore, it was decided that additional participants in the intervention arm be invited for an interview at four months and twelve months to increase the diversity of our sample.

We purposively sampled both male and female participants with a range of ages and locations across different classifications of chronic headache. We sent this sample a participant information leaflet detailing the reasons for doing the interviews. Those who wished to participate were then booked for a face-to-face interview in their choice of convenient location (home, work, local community room). We used semi structured interview topic guides. (see supplementary material 1, pages 1–4) This was adapted/updated using our learning from the phase one interviews. Interviews asked about their lived experience of their headaches and different aspects of the impact of this on their quality of life.

### Data collection and processing of all interviews

All interviews were audio-recorded on an encrypted digital device (OLYMPUS DS-7000), kept in a digitally safe environment, given pseudo identifiers before being transcribed by a member of the team who removed any identifiable data from the text. The researcher who did the interviews (VN) then checked the transcripts for accuracy. Researcher notes were written immediately after each interview to capture aspects which may not have been apparent from the recording, such as body language and the emotional tone of the interview as well as the researcher’s views, to promote researcher reflexivity and cross case comparison. We used NVivo software to manage and organise the data.

### Analysis

For Phase 1 interviews we used thematic analysis [13], structuring the themes by the experience of interviewees from becoming aware of a headache through decisions about what to do in response to the headache. Two researchers (VN, FG) read and familiarised themselves with the transcribed text, developed codes, discussing, and updating the codes as analysis continued (VN, FG & DE).

In Phase 2 we had learnt from the phase one interviewees that how they experienced their headaches changed over time. Our focus was on exploring this change over time using serial interviews, e.g., those who gave interviews at baseline, four months, and 12-months. To do this, we brought into our analysis only data where the participant talked about their current headaches. For each interview we wrote a pen portrait. Pen portraits are a way in which longitudinal health research data can be concentrated into a focused account [14]. The methodology is a four stage process: i) understand and define what to focus on; ii) design a basic structure relevant to the dataset in question; iii) populate the content; and iv) interpretation. Stages one and two were informed by the data emerging from the phase one interviews providing a structure with the following five headings: context, different headaches, medication management, non-medication management, and quality of life. Initial pen portraits were reviewed and refined by the team until we established a consistent approach. Pen portraits were compiled by VN.

The team read the pen portraits and developed categories, a method we have previously used to identify change in categories of experience with pain over time [15]. These categories can be considered as ‘categories of headache impact’ whereby participants may match mostly with one category but have characteristics of other categories to varying degrees [16]. Each team member independently categorised a third of the pen portraits with VN categorising them all. Subsequently meetings were held to compare categorisations, discuss any discrepancies, to agree a final category for each pen portrait and give the categories titles.

The final set of interviews included participants recruited within the intervention arm of the trial at four months and again re-interviewed at 12-months (as noted in the methods above). Four additional participants (one intervention arm; three control arm) were interviewed just twice—at baseline and four months – and are included in this group. Again, pen portraits were crafted (as detailed above). However, as a validation check, the categories defined in the previous group analysis were applied to check if they resonated across this subsequent sample.

## Results

### Phase 1: Initial interviews

We interviewed 15 participants (10 female) age range 29 to 69 years (median 47 years): two had chronic tension type headache, six chronic migraine, and seven probable chronic migraine. Additionally, six of the 15 had medication overuse headache. Participants lived across a range of localities in the English Midlands: three rural, eight in towns and four in a city (Table 1).

**Table 1:** Phase 1: Interviewee characteristics

**Int ID**	**Location**	**Sex**	**Age**	^**a**^**Headache** **Classification**	**Int ID**	**Location**	**Sex**	**Age**	^a^**Headache** **Classification**
LE2	Rural town	F	45	DCM + MO	LE10	City	M	47	PCM-MO
LE3	City	F	29	PCM-MO	LE11	Town	F	67	PCM + MO
LE4	Town	M	65	DCM + MO	LE12	Town	M	69	CTT-MO
LE5	Town	F	54	DCM + MO	LE13	City	M	69	CTT-MO
LE6	Rural town	F	45	DCM + MO	LE14	Town	F	51	DCM-MO
LE7	Rural Town	F	42	DCM-MO	LE15	Town	F	35	PCM + MO
LE8	Town	F	51	PCM-MO	LE16	Town	F	44	PCM-MO
LE9	City	M	30	PCM-MO					

^a^DCM +MO ‘Definite chronic migraine’ and medication overuse headache*. PCM* + *MO* ‘Probable chronic migraine’ and medication overuse headache, *DCM-MO* ‘Definite chronic migraine’ without medication overuse headache, *PCM-MO* ‘Probable chronic migraine’ without medication overuse headache, *CTT-MO* ‘Chronic tension type headache’ without medication overuse headache

Medication used by participants was, on the whole trial and error from over the counter (OTC) medicines at the outset. If these didn’t work then they would seek help from their GP for suggestions of other medications to try. The OTC medications used at the time of interview ranged from paracetamol preparations/ ibuprofen/, and opioid analgesics most of which helped but didn’t abolish the headaches. A few were on preventatives such as pizotifen, beta blockers or amitriptyline prescribed by the GP or a neurologist. Triptans were also prescribed which were useful for some. Others used medication prescribed for other conditions for their headaches such a naproxen, propranolol, or codeine. Around half described having tried preventatives in the past which were mostly unsuccessful.

Our participants recounted often long complicated medical/ personal/ headache histories. Most talked about experiencing different headaches, dichotomising them as ‘normal’/ ‘not severe’ or ‘migraine’/ ‘severe’ headaches. At onset, sometimes they were aware immediately which headache type it was but sometimes they had to wait to see how it progressed before identifying the headache type, gauging its intensity or its nature. The presentation and the context of the headache were important considerations as to what they would then do in terms of taking medication, altering plans, considering how it would impact others. Sometimes they had to leave their current environment because of the headache. Decisions would depend on whether they were at work, home or out somewhere and whether they felt they could manage the headache where they were. Participants described an often-continual search for triggers or an acceptance that there were no identifiable triggers. They described how the experience of headaches impacts on their lives including the frustration at people not understanding how it affects them and how fed up they were with having headaches. Table 2, provides illustrative quotes of their experiences of chronic headache.

**Table 2:** Phase 1 interviews, showing themes and illustrative quotes

Phase one lived experience themes		
**Theme**	**Subthemes**	**Exemplar quotes**
**Nature of the headache** (Different headaches)	**A.** Not so intense Ignore Take meds Keep going	*And then in-between that I get… well I just I refer to as a headache a general everyday headache… mmm… sort of woken up with one this morning and I think… it hasn’t got the intensity to it, it’s just a little ‘niggle’ so to me that’s just a normal headache…LE8* *because I get rid of them if they’re a problem… …and they don’t impact…* *…and you know it would be uncomfortable to go through the day with one I suspect but I’m not sure, you know, I wouldn’t be taking to my bed or necessarily not moving things but I would get less enjoyment from the things that I did I think…LE12*
	**B.** Intense Unable to ignore / wait until sure it’s a ‘bad one’/ Keep going up to a point where unable	*‘I’ve got a headache today, but it’s not my normal headache’……L4* *It is the intensity of it, it is the crushing feeling that I get at the front of my head… mmm… sort of left and right around the temple area as though somebody has just got their hands either side and they are attempting to crush my head and then it might settle in a particular area…LE8*
**Gauging:** (and taking action to try to keep functioning)	**A.** Perception of the type of headache	*but I just generally ignore them, I’ve had them so much it’s just a part of life*…*… it’ll start and I’ll think “oh it is just a headache” and go and grab some tablets, you know, normal Paracetamol or Ibuprofen something like that whatever I’ve got to hand but within half an hour or even 20 min you know that it’s… it’s not because it’s just so intense LE8* *… while it’s at full swing and just keep taking tablets and drinking water that’s all I can do really…LE9* *Yeah and I’m talking to you now and I know I’ve woke up with one but I can deal with this one it’s fine it’s not affecting me at all……LE16*
	**B.** Whether early medication is needed	*… this morning I woke up with a headache roughly about a 6 to a 6.5 and I thought ‘I think I am gonna take something’ mmm… because you were coming and I thought I don’t want it to sort of like, you know, for me to be not… not being able to concentrate that much so but… but you know normally I wouldn’t’ve taken anything but if it gets to a 7 then I will take something definitely.LE5* *I am starting taking Imigran occasionally mmm… which… which does work but it does knock you out. So I can’t… it’s not something I can take if I’m going to work or, you know, I’ve got to it… I sort of save it for specific times… LE6*
	**C.** How bad will it get with or without meds?	*If I take medication early enough I can hold it at bay mmm… so that I don’t have to go in a darkened room and everything, but they will just keep coming back until I’ve actually gone and been in a room by myself for a b*it*. LE6* *and I do still occasionally use those but again it… it’s in the vain hope that they will help but in fact, I take them and think “ok I’m still feeling bad, but would I feel worse if I hadn’t taken them!” LE8* *sometimes if I get up fairly quickly it will gradually within the next hour go off but if it’s not going to go off then it will last and then get slightly worse… slightly worse until I think ‘oh no this isn’t going off’ I’ll go out outside and, you know, clean the hedgehogs out and try and get in the fresh air and think ‘mmm… you know I’m feeling alright’ and then I’m thinking ‘no this is not going!’LE11* LE5
	**D**. What has triggered this headache?	*‘working this out’ LE7* *I’m constantly looking for ‘why’ and what I’m doing…LE6*
	**E**. When to keep going and when to stop	*‘…it’s kind of disturbing the time do I do this or stop things which I’m doing at the moment it’s not that bad yeah I’m not a child, I’m not going to be, you know, going to the corner and crying about that but it’s just really disturbing when I’m at work I’m going through the aisles and I’m like ‘ah I can’t focus on this……it’s just pissing me off! LE3 LE8*
	**F**. How long will it last?	*… whether it’s a headache that lasts an hour or six hours or 10 h, it’s just one of those things I just can’t tell! LE8* *Yes so if it hasn’t gone within two hours then I know it’s not going to go without… err… a helping hand! LE11*
	**G**. How it impacts on work. Link with presenteeism	*…I need to be able to concentrate in my job ‘cause the work I do (can halt the production process) and cause millions of pounds of damage or problems, so I just have to ride it out really maybe I’ll slow my work pace down…* *… it’s not very often I have to walk away from my desk but occasionally I might have to say to the guy next to me “look I need ten minutes! ……I refuse to give in to a headache to come to work… LE9* *but I have to go to work I can’t just stop going to work… stop going to work because I need the money and so I’ll move things and I’m going to go to work anyway but I do it slower…LE3*,
	**H**. How it impacts on social activities -Altering or cancelling engagements	*it’s really only in the last 2 or 3 months that I’ve got to the stage of, you know, I’ve got to accept that it’s a migraine. You know, ‘bang’ goes my work life, and ‘bang’ goes my social life!LE8* *the times I think when I have had a migraine and you know you are meant to be going out with friends for a meal or going for a party, you know, you can sometimes guarantee if you are going out you’d always get some sort of migraine…LE14* LE4, LE11
	**I**. How it impacts on others?	*And you’d think everybody’s looking at you, but they can’t hear you …… they can’t hear what you can hear in your head, LE2* *‘Tuesday’ he’d find me laid out on the sofa……saying “oh are you alright?” “no I’ve got a migraine!” he said like “ok then” you know, alright, it kind of frustrates him I think because he can’t do anything to help me… mmm… and then sometimes I was supposed to be going out on Tuesday evening and it’s just, ‘nope I can’t do it!’ LE8*
**Headache taking over -** Gets to a point where headache determines **Flexibility of context**	**A**. Inability to function Need to ‘shut down’ or sleep	*But the migraines, if I’m at work, I just have to finish off whatever I’m doing… tidy up what I’m doing and get home because to drive… for me to drive with a migraine, I just feel so sick with it, movement is my thing…… if I’m feeling particularly bad I’ll come home and go to bed and try and sleep through it…LE8* *… it stops you doing what you normally want to… just normal day to day chores because you just feel too poorly to do anything…LE14*
	**B.** Avoidance of aggravating environment	*… I might sometimes have to have a ten minute get away from the desk because, if there’s a lot of background noise going on as well…… I will put my headphones in to try and block out noise … LE9*, *… I stopped going to Church because of the band at Church and the noise of people…LE15*
	**C.** Need to be (or feels) sick	*but when I do get that feeling of sickness it’s almost like you think I just want to go and lie down, I feel really sick, I’ve got to be sick, I can’t go to sleep until I’ve been sick. It’s really gonna hurt if it gets any worse and I’m sick… so in the end I make myself sick just so that I can ‘done that’ and I can go to bed, put the compress on, blacken the room and everything and just, sort of, shut myself off.* LE6 *When they get really, really bad I feel sick, I don’t want to eat, umm, and it’s like your eyes just shake.……, it’s not going dark but it sort of like closing in, like really want to shut your eyes…LE2*
	Able to manage headaches due to flexible context or not	*‘cos I do work for myself so I can swap and change a lot of things you see…… If I had a 9 to 5 job, I don’t know how I’d cope. LE2* *… but it just, sort of, get more intense at times that’s… that’s… that’s how… sometimes it’s… it’s… I can go… I can go… especially something like working in the garden where I’m, you know, I… I… I don’t go like a bull at the gate but if you… you do an hour here and an hour there but you can keep the garden quite nice and tidy and attractive and I try to do that…LE13* *…I do do things on the Friday I tend to have quite a lot on Friday but socialising and different things but Saturday I don’t tend to do anything and Sunday… well I do my housework or I might have someone round for a meal but I always take it easy because I know I need to rest for going back to work on Monday… …and I don’t know whether I’d ever… at the moment whether I’d be able to cope with a five day week…LE15* *So if I was in a different kind of 9 – 5 very structured role, I can’t imagine it would be a nightmare! It would be a nightmare but I’m very lucky that I have somebody who is… who is understanding and as I said we don’t… I don’t even register them as sick days, I register them as ‘working from home’ ‘cause he knows that at some stage during the day when I’m feeling a bit better I’ll have a look at my emails and I’ll deal with the most urgent things, I stay on top of everything. So yeah, I’m very lucky in that regard. LE9*
**Triggers**	**A**. Known or suspected e.g. Weather, Hormones, Alcohol Sunlight, Work environment, Posture, Dehydration, Perfume, Stress, Flying, Lack of sleep and certain foodstuffs	*because my triggers, you know, might not be their triggers…LE7* *… I think it is just a lot of contributing factors really but stress, I would say, is probably a big factor.LE9* *but I know it’s there… it’s got a slight dull… I can feel it in here… it’s here. But that I now know, I’m 100%… well I know my neck’s sore… my neck’s very, very tender the last few days I don’t know what I’ve… humping boxes doing Christmas presents probably… mmm… and the sweating that I’m having through the night is definitely the lack of water.LE16*
	**B.** Unknown	*Well it just does it when it wants to do* it…*LE2* *‘hard to pin point’ LE9* *…and there seems to be no difference at all really, I mean in… in terms of diet, alcohol intake, coffee, exercise I mean it just… it just sometimes I get it and sometimes I don’t. LE12*
**Emotional impact**	**A.** Not being believed	[at GPs] *I cried, and I went “just because I look alright, and I’ve got my make-up on and I’ve washed my hair, it doesn’t mean it is alright”.* LE2 *…I just want them to understand……how ‘rubbish’ it makes me feel…..I always think that people think… … it’s like “oh everyone has headaches, deal with it!” LE6* *… some people who don’t ever suffer with headaches say “well I don’t take anything for a little pain like a headache!” I could, (laughs) you know, throttle people like that really…LE11*
	**B**. Worries	*It’s scary at nighttime…* And so we’re going out in a couple of weeks’ time and I don’t worry about, about it, but……It does cross my mind cos I hope it, I hope me head’s alright and I’m not grumpy and stuff *LE2* *…so you do get a little bit worried at times, ‘is it gonna to strike when you don’t want it to’ it’s just little things like… even at things like Christenings, you know, people playing music everyone is having a good time when you are sitting there and your head’s throbbing*… *then I’ve worried thinking what kind of headaches this, is this the classic migraine or is it not …LE9*
	**C**. Irritable, irritated	*…I take it out on my girls and family, you know, my husband… I can’t help it because you do feel irritable, you do feel miserable ‘cause you just don’t feel well… whereas if you didn’t have that pain you wouldn’t feel like that… LE14* *…it’s just pissing me off ! LE3*
	**D**. Depressed, Can’t cope	*… I felt depressed for myself extremely sorry for my husband I mean it’s not easy putting up with somebody who… whose… whose got… well I was completely useless, couldn’t do a darn thing when I was… when I was suffering like that and… and also after a while I think people loose sympathy because there’s no sign of… if you’ve got a leg in plaster “oh, oh what a shame that must be painful!” but, you know, apart from looking half dead… mmm… there’s no sign really LE11* *… well it prevented me going out to see my friends it prevented me… mmm… it’s… it depressed you I think a little bit you go in your inner self with it… LE15*
	**E.** Decreased enjoyment / spontaneity / guilt	*…in a month’s time, if I didn’t feel like going cos of my head I wouldn’t go……You know even though I’d probably be really looking forward to it, seeing family and things like that……I wouldn’t go if it was my head……No. I would sit there miserable, so there’s no point doing that…LE2* *…my Mum said what she remembered from it[a nice holiday experience] was that I had a migraine and that they needed to get back…… it just makes me feel really guilty about things, LE6*

### Phase 2: Main Study interviews

#### Comparison across all time timepoints (developing and testing the categorisations from the pen portraits)

A total of 26 participants were interviewed, from a variety of locations in the Midlands or London. (Table 3) These 26 participants make up the two groups below.

**Table 3:** Phase 2: interviewee characteristics

						**Interviews**		
	**ID**	**M/F**	**Age group**	**Trial arm**^**a**^	**CHESS Headache classification**^**b**^	**Baseline**	**4 Months**	**12 Months**
**Developing the categorisations from the pen portraits**	**1**	F	40s	Int	DCM + MO	*	*	*
	**4**	F	20s	Con	DCM + MO	*	*	*
	**6**	M	20s	Con	PCM-MO	*	*	*
	**7**	F	70s	Con	PCM-MO	*	*	*
	**8**	F	60s	Int	TTH-MO	*	*	*
	**9**	M	60s	Int	DCM + MO	*	*	*
	**10**	F	50s	Int (dna)	PCM-MO	*	*	*
	**11**	M	50s	Con	PCM-MO	*	*	*
	**12**	M	40s	Con	PCM + MO	*	*	*
	**14**	F	20s	Con	DCM-MO	*	*	*
	**15**	F	50s	Int	DCM-MO	*	*	*
	**17**	F	50s	Int	PCM + MO	*	*	*
	**19**	M	70s	Int	PCM-MO	*	*	*
	**20**	F	50s	Con	PCM + MO	*	*	*
**Testing the categories**	**02**	F	30s	Con	PCM-MO	*	*	
	**05**	F	20s	Int (dna)	DCM + MO	*	*	
	**16**	F	50s	Int	DCM + MO	*	*	
	**18**	F	50s	Con	DCM-MO	*	*	
	**22**	M	30s	Int	DCM-MO		*	*
	**23**	F	30s	Int	PCM-MO		*	*
	**24**	F	50s	Int	PCM + MO		*	*
	**25**	F	40/50s	Int	PCM-MO		*	*
	**27**	F	60s	Int	PCM + MO		*	*
	**28**	F	50s	Int	PCM-MO		*	*
	**30**	F	30s	Int	PCM-MO		*	*
	**31**	F	40s	Int	DCM + MO		*	*

^a^*Int* Intervention arm, *Con* Control arm, *Int (dna)* did not attend any intervention elements

^b^*DCM + MO* ‘Definite chronic migraine’ and medication overuse headache, *PCM + MO* ‘Probable chronic migraine’ and medication overuse headache, *DCM-MO* ‘Definite chronic migraine’ without medication overuse headache, *PCM-MO* ‘Probable chronic migraine’ without medication overuse headache, *TTH-MO* Tension type headache without medication overuse headache

Fourteen (median age group 50s, range 20s – 70s; 9 females) were interviewed three times (baseline, 4 and 12-months; total 42 interviews). Participants were randomly assigned to control (*n* = 7) and group intervention (*n* = 7) arms of the trial. However, one participant did not engage with the intervention (did not attend DNA) and was classified as ‘control group’. These interviews informed the development of pen portraits and proposed impact categories.

Twelve participants were interviewed twice (total 24 interviews; median age group 40s-50s 11 females). Four were interviewed at baseline and four months, three of these were in the control arm, with one in the intervention arm (but they too did not attend any intervention elements DNA). Eight were interviewed at 4 and 12-months (age etc.; 7 female); all were in the intervention arm. These interviews were used to test the impact categorisations.

The developed categorisations from the pen portraits (From the dataset of the 14 triple interviews).

**Fig. 1:**
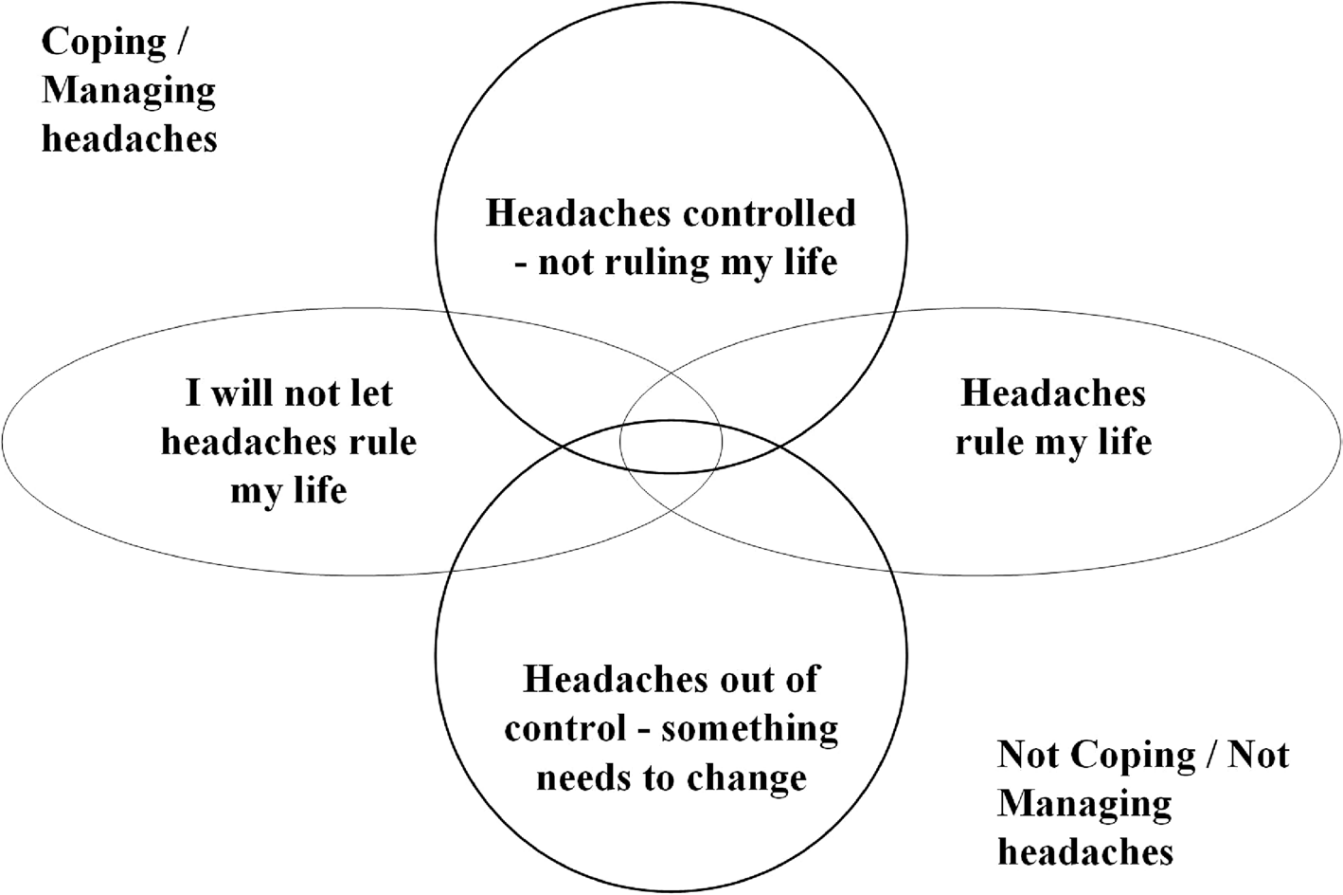
Concept model of the relationship between categories of headache experience

Figure 1 above shows the four categories developed from the longitudinal interview data. It was very clear that as time goes by people often change their categories and sometimes exhibit characteristics of some of the other categories at the same time hence the diagrammatic overlap and close relationship between all of the categories.

To present our findings we have written short summaries of the pen portraits for each participant and presented these as a table of how they were categorised at each time point.

Tables 4 and 5 below show summary pen portraits from each of the interviewees who gave interviews over the three timepoints (Baseline, four and twelve months). Each participant at each timepoint is coded with one of the four categories. Examples of actual pen portraits, illustrating each of the four categories, are provided in supplementary materials. (see supplementary material, pages 5–21).

**Table 4:** Headaches over time – Summary pen portraits at baseline, 4 ms and 12 ms (CHESS Control participants)

ID	Baseline	Cat	4ms	Cat	12ms	Cat
4	**Headaches rule my life** Life severely affected by headaches	II	**Headaches controlled—not ruling my life** Pregnant. Headaches slightly better, less severe eye symptoms. Only able to take paracetamol	IV	**Headaches controlled—not ruling my life** Although headaches worse after the birth now feels ‘stable’ but would like to find other non-drug treatments	IV
6	**Headaches controlled—not ruling my life** Doesn’t let it impact on their life	IV	**Headaches controlled—not ruling my life** Same no change	IV	**Headaches controlled—not ruling my life** Same	IV
7	**Headaches controlled—not ruling my life** Doesn’t stop them from doing things. Able to work around some due to retirement. Headaches better now than when working. Has confidence in management. Settled	IV	**Headaches controlled—not ruling my life** Same	IV	**Headaches controlled—not ruling my life** Headaches maybe less frequent needing to take less medication attributes to getting older	IV
10	**Headaches rule my life** Living a smaller life. Unable to plan prioritises work but social life pays for it	II	**Headaches rule my life** Although headaches may be slightly better effects are the same as baseline	II	**Headaches out of control—something needs to change** *The plan is I’m not going to give up… the plan is we keep trying new things… the plan is that every article or anything I see or any treatment… I’m not saying any treatment because people say ‘oh you must try my chiropractor you must try my osteopath they’re amazing’ and it’s like I’ve done all of that…*	III
11	**I will not let headaches rule my life** Doesn’t stop them from doing things	I	**Headaches controlled—not ruling my life** Feel they are managing headaches better less frequent	IV	**Headaches controlled—not ruling my life** Reached a point of acceptance self-efficacy and confidence in managing their headaches	IV
12	**Headaches rule my life** Life governed by headaches. Spends 2/3 days a week in	II	**I will not let headaches rule my life** Getting out more in spite of headaches, managing headaches better. Medication same	I	**Headaches controlled—not ruling my life** Managing headaches better, taking less medication, feels more positive	IV
14	**Headaches rule my life** Unable to plan spoils life when gets them. Severe stops life	II	**Headaches controlled—not ruling my life** Context and severity dictating management. Headaches less severe (pregnant)	IV	**Headaches out of control—something needs to change** Headaches worse uncertainty and stress of new job. Return of severe headaches after giving birth	III
20	**I will not let headaches rule my life** Headaches differ in severity. Dizziness and tinnitus a feature Bad headaches will stop them from socialising Manages their headaches from just putting up with them to taking medication (paracetamol and preventatives). Indian head massage helps. When bad they will lie down, relax and try and sleep it off. Uses a sound machine to manage tinnitus	I	**I will not let headaches rule my life** Headaches better Tinnitus still a feature but not as bad. Keeps track of them with a diary, ‘rides them out’ using medication as necessary *I ride them out basically yeah… …they’re generally not too bad at the moment they seem to be behaving!* *… but yeah as far as my headaches are going I’m sticking with the medication and just keep taking the tablets… not a lot else I can do really… but yeah it’s what you have to live with ain’t it unfortunately*!	I	**Headaches controlled—not ruling my life** Attributes decrease in headaches to being on right meds (increased nortriptyline) Also relatively stress free time *They’ve actually been getting better a lot… lot better… mmm… if you look at my diary…* *… no but I’m quite… at the moment I mean they’re… they’re quite settled and I’m quite happy the way they’re settled*	IV

*Cat (categories) I = I will not let headaches rule my life, II = Headaches rule my life, III = Headaches out of control—something needs to change, IV = Headaches controlled—not ruling my life

**Table 5:** Headaches over time – Summary pen portraits at baseline, 4 ms and 12 ms (CHESS intervention participants)

ID	Baseline	**Cat**^**a**^	4ms	**Cat**^**a**^	12ms	**Cat**^**a**^
01	**I will not let headaches rule my life** Ok as long as has medication says it doesn’t interfere with life (although it does at times|)	**I**	**I will not let headaches rule my life** Nothing’s changed in management or medication	**I**	**I will not let headaches rule my life** *I don’t think I’ve got a plan at the moment for doing anything particularly different because it’s manageable although it’s not particularly pleasant*	**I**
08	**I will not let headaches rule my life** *‘It slows you down a bit sometimes and you feel that, ‘ oh I can’t be bothered to do that…I’ll leave that to another day’…otherwise I just try and get on with life in general…*	**I**	**I will not let headaches rule my life** Headaches were worse due to neck problems then better after saw physio. Headaches drinks more water takes more ‘me time.’ Trying mindfulness	**I**	**I will not let headaches rule my life** Main change is stress due to family needing support. Everything else the same. Considering getting help with unhelpful thinking	**I**
09	**Headaches rule my life** Life affected by headaches, worried when has more headaches or more medication	**II**	**I will not let headaches rule my life** Optimistic about new preventative tablets	**I**	**Headaches controlled—not ruling my life** Headaches have decreased bringing increased confidence*.… what I’m trying to do now is push all that into the background and not even think about them and not even think, ‘Oh will I have a headache!’ or you know ‘Am I gonna have a headache?’ or maybe’ I won’t do this cos I might get a headache!’ … that is a bit easier you know when you have less headaches anyway … because there’s very few entries this year compared to the last two years it does you know enable me I think to sort of… yeah just try and sort of forget about it and… mmm… just carry on almost you know as normal as though nothing’s happening and not trying to manage my life around you know the possibilities that I might have a headache or something like that*	**IV**
15	**Headaches out of control—something needs to change** Headaches affecting all areas of life severely	**III**	**Headaches out of control—something needs to change** Headaches still as bad, mood has been worse, psychiatrist has added an anti-depressant, neurologist has changed triptan due to tiredness	**III**	**Headaches controlled—not ruling my life** Mood much improved by addition of an anti-depressant. Looking at ways they can be more in control and do more activities using tools taught at the intervention. Acceptance, unhelpful thinking, altering lifestyle by drinking more water and accepting more social engagements	**IV**
17	**Headaches rule my life** Headache rules their life but has caring responsibilities which also impact on their QOL	**II**	**Headaches rule my life** Headaches similar, life similar	**II**	**Headaches out of control—something needs to change** Not under control, stressful circumstances worsened looking after relative with learning disabilities. Taking more medication headaches worse	**III**
19	**I will not let headaches rule my life** Even though they have regular headaches they doesn’t let them stop what they’re doing, uses distraction and gets on with their life. Feels they would experience their headaches more if they focussed on it or talked about it	**I**	**I will not let headaches rule my life** Similar to baseline	**I**	**Headaches controlled—not ruling my life** Adding a triptan has helped them to control their headaches a bit more *I mean because I know it’s gonna to be pretty ok after about an hour and then I can go out and if it delays it until the following day I prefer to do that cos you know I can enjoy when I’m going out… so it is… they are better and certainly more manageable and it makes it more flexible if you know what I mean*	**IV**

^**a**^Cat (categories) I = I will not let headaches rule my life, II = Headaches rule my life, III = Headaches out of control—something needs to change, IV = Headaches controlled—not ruling my life

Table 4 shows interviewees who were not exposed to the CHESS intervention. At baseline four interviewees were categorised as their headaches ruled their lives, two whose headaches controlled – not ruling their lives, two were not letting headaches rule their lives and none were categorised as Headaches out of control. Six of the eight interviewees changed category at least once over the twelve-month period.

Table 5 shows the interviewees who were in the CHESS intervention arm. At baseline three interviewees were categorised as not letting headaches rule their lives, two where their headaches ruled their lives, one where they were catergorised as headache out of contol and none were categorised as headaches controlled – not ruling their lives. Four of the six interviewees changed category at least once over the twelve-month period.

### Relationship between categories

Whilst a predominate category is defined for each interviewee at each timepoint it was very clear that many exhibited elements from others demonstrating the complex nature of living with chronic headache. For example, interviewee #08 (Table 5) who is not letting headache rule their life but also notes that at times they put things off because of it. Whichever the predominant category we decide on there are elements of another category. Some people are at the boundary of a category, so #09 at Timepoint 2 is categorised as not letting headaches rule their life but bordering on their headaches being controlled which by Timepoint 3 they are.

Those sitting in the upper left quadrant of the conceptual model are more likely to be managing with the strategies they have, with headaches impacting little on their day-to-day functioning. Those sitting in the lower right quadrant are experiencing headache effects which either limit or dominate what they can and can’t do.

### Validating the categories (interviews at two points in time)

Table 6 Provides summaries of the pen portraits from the twelve pairs of interviews. We were able to categorise participants at each time point using the proposed categories, and as before, some participants expressed elements of another but less dominant category. Of the four who gave interviews at baseline and four months two, at baseline, were categorised as their headaches were ruling their lives and two as their headaches were out of control and something needed to change. At four months all were no better and indeed all were categorised as their headaches were out of control and something needed to change. Of the eight who gave interviews at four and twelve months: three, at four months, were categorised as not letting their headache rule their lives, three were categorised as their headaches were ruling their lives and two were categorised as having headaches that were controlled. At twelve months all but two of the eight participants changed categories, with two re-categorised as ‘headaches out of control’ and four as their ‘headaches are now controlled’. The final two are categorised as, not letting headache rule their life and headaches not ruling life.

**Table 6:** Summary pen portraits testing the categories defined earlier in pairs of interviews (two timepoints)

ID	Baseline	Cat	4ms	Cat	12ms	Cat
02	*I think it’s called an aura when you get like a feeling that’s it’s going to come on if I catch it at that stage and take some tablets and rest and things I can normally at the minute kind of ‘curb’ it I think work a lot of it is the problem because the lights are so bright…*	II	*I think so yeah… I think it’s the stress and panicking about stuff over the last two months particularly so I’ve had quite a bad headache quite a lot, so I think it is* *probably due to that*	III		
05	*I’ve got a headache! (inaudible). It changes a lot some days I feel like I could die! I feel like you know what, I wanna scream because that’s how much agony I’m in and then it’s that, the fact that, I had to …in the past three days. It’s got really bad (inaudible) I want to do something I don’t want to be here no more I want to end my life (inaudible) so it brings me down as a person as well*	III	Complex, lots going on in life mental health not at all good. In a bad place not really managing it	III		
16	*The thing is with headache… with the headache I can’t say that ok I’m going to get a headache at 10am I won’t do anything in-between that space but I… it just doesn’t happen like that!*	III	…whereas it doesn’t seem to matter what you do it *just keeps happening and it’s all the time… all the time… yeah I find it more probably more stressful in that I’m not find this conversation stressful but things to do with… important stuff am not very good at, I kind of lose my way… mmm… err… I don’t… I can’t think of anything else that I’ve… can do to alleviate it… I do know that I tend to* *not get any sleep at night so I’m not waking up early in the morning and that’s nearly every bloody day it’s awful you feel like you’ve lost half the day…* *… I don’t really take the tablets anymore I try to avoid it and use a… I’ve got a cold compress that I keep in my fridge all the time I put that on and I’ve got the things that you cover your eyes with when you go to sleep…*	III		
18	Elements of out of control Takes medication but not managing. Impacts on all areas of life	II	Worse. lots of other health problems even tablets don’t work anymore *As I’m talking to you right now is like the pain shooting right up in me head right here so you know I can feel the spot right here so the pain… I told you!*	III		
22			Impacts on whole life *headaches every single day you know full on migraines every day sometimes full-on paralysis… blindness… mmm… I get all of that every day… mmm… again the GP’s like ‘well that’s not how it’s meant to go you’re meant to have a (inaudible) and then… so there’s processes you are meant to go through which is confusing as to why it’s every single day*	II	Impacting more on life *think I’m getting to a point where I’m trying to sort… mmm… demotivated to try and find a solution I’m feeling like I’m accepting defeat and I’m just going to have to live constantly on painkillers and just have to suck it up and get on with it… I… I… yeah I just feel defeated with it all in all honesty!*	III
23			*Not sure why I’m getting so many all the time and it would be nice to not get them all the time but why… why stress over something that you can’t change when you’ve got plenty of other stresses so yeah from now on it’s a case of just make sure that whatever I do I’ve got painkillers within reach and just carry on… battle on through!*	I	Impacting more on life *think I’m getting to a point where I’m trying to sort… mmm… demotivated to try and find a solution I’m feeling like I’m accepting defeat and I’m just going to have to live constantly on painkillers and just have to suck it up and get on with it… I… I… yeah I just feel defeated with it all in all honesty!*	III
24			*I suppose it’s because I’ve managed all my life… I’ve been suffering from headaches since I was about 12 so roughly roundabout 12 so and it’s just something that you’ve grown used to… mmm… medication… mmm… unfortunately I’ve got the stage where I… I know that I’ve got medication overload (laughs)…*	IV	Some deterioration *I don’t know really I mean I’ve tried to you know I keep going back to the Chiro and everything but obviously I don’t really know if that actually works so I don’t know what to try next because I’ve been down the roads and other routes and it all helps a bit and then it* *doesn’t so!*	II
25			*I think the one thing that is better is my mind-set and you know I’m even more in that acceptance space so that’s… the course just reinforced my belief that I’ve done everything I can to what I call kind of get rid of the headaches and I pretty much know what I need to do to manage them and if they get worse I know what to do about that too… mmm… so you know in a kind of reassurance sense it has been helpful!*	IV	*the headaches have been pretty much the same as usual and so I have a headache every day more or less I would say with the odd day with no headache every couple* *of months and then I have a kind of worse sort of migrainey slightly more debilitating headache probably one a month or so* *So I guess I’m pretty used to it now so I just kind of accept it and move on (laughs) so obviously to keep them in check I’m on the Venlafaxine as a Prophylactic which seems to be helping quite a bit and then I try and make sure I get enough sleep and I am not dehydrated* *and that my blood sugar stays stable you know all the kind of good advice…*	IV
27			*my migraine don’t appear to be as severe as them I don’t have to go and lie down… I carry on because I can and with the medication 98% of the time the medication for me means that I can carry on… well I do carry on anyway so it’s not as bad as some of the people… it’s unpleasant but I carry on*	I	*… mine aren’t that bad I mean they’re not very pleasant but they don’t stop me doing things… mainly because the Sumatriptan does work generally it certainly takes the edge off the pain… if it doesn’t… if initially it doesn’t take away altogether which sometimes it doesn’t it does take the edge off*	IV
28			One might feel that this person is not letting the headache rule their life but on reading their narrative it is clear that it impacts considerably. They do have some control with the medications but there are very intense periods where the headache wipes them out and stops them doing everyday things	II	*Mmm… I have been able to control them… I have them mainly at the weekends and I’ve managed to work through they’ve not been too bad… not as bad as they used to be but I’ve had them now… I’ve been feeling tense you know… just tensing up and I’ve been able to try and relax myself kind of thing but saying that over the last couple of months I’ve had that flu virus that’s been going round…*	IV
30			*So I get them most days… mmm… most of the time they’re just sort of in the background so most of the time I can just get on with my jobs and everything… mmm…* *most of the time it’s just sort of somewhere on the left-hand side where it just sort of niggles a little bit… mmm… pain levels sort of one to three… mmm… but sometimes they can get progressively worse throughout the day and sometimes they get really bad that I just think I’ve got to go to sleep or got to have time off work and I can’t cope with daily functions…* *yeah which is pretty bad!* *…just trying to find the balance of my tablets…*	II	*They’ve been much better actually since I’ve been on the Propanolol… mmm… I* *haven’t had… yeah I haven’t had any sort of migraines I’ve only had sort of little headaches* *and that’s usually when I haven’t taken my tablets when I should so I know that 'oh actually I* *probably need to take my tablets' so that’s really good*	IV
31			They’ve been much better actually since I’ve been *on the Propranolol… mmm… I* *haven’t had… yeah I haven’t had any sort of migraines I’ve only had sort of little headaches* *and that’s usually when I haven’t taken my tablets when I should so I know that 'oh actually I* *probably need to take my tablets' so that’s really good*	I	*I just put up with it! … I mean I take Paracetamol and Ibuprofen if it’s bad otherwise I’ll just put up with it… cos you… I mean I’m used to it… it’s almost like you sort of forget you’ve got one because you’re so used to having one…*	I

^a^Cat (categories) I = I will not let headaches rule my life, II = Headaches rule my life, III = Headaches out of control—something needs to change, IV = Headaches controlled—not ruling my life

All four categories could be influenced by many external contextual factors e.g. if they were retired, if they were able to work around their headaches, where they were when a headache started. Where one person had to battle through another had the flexibility to take time out for it to ‘work through’ with or without medication. Others kept going even with severe headaches that others wouldn’t have been willing or able to do.

## Discussion

All participants involved in these interviews attested to meeting the CHESS study criteria for chronic headache defined as headache on 15 or more days per month for at least three months with most reporting these as migraine. In a pre-randomisation telephone interview, we classified just over half as having chronic migraine. The phase one interviews revealed that their headaches had often developed over long periods of time and were embedded into their lives giving a complex fluctuating presentation. Headaches appear to determine how people can function in their everyday lives and a great deal of time and energy is spent anticipating the severity of the next headache and gauging effective strategies such as medication use and behavioural approaches to lessen their severity and impact. Different headaches can feature as a difference in severity or nature which can alter the strategies used.

Throughout we see that people are often struggling to function and ‘keep going’ most often in flux and dependant on many factors. This complex picture keeps people gauging what to do, when and how to do it whenever a headache presents, often learning from trial and error.

What we see in these results is the often overwhelming and comprehensive effect that living with this condition has on people. Our pen portraits illustrate this complex picture demonstrating that these are not marginally troublesome issues rather they are something at the heart of health, well-being, and quality of life. Our findings are similar to those of a study in Spain looking at the views and experiences of women living with chronic migraine [17]. They identify five key themes: i) the shame of suffering from an invisible condition; ii) treatment: between need, scepticism and fear; iii) looking for physicians’ support and sincerity and fighting misconceptions; iv) limiting the impact on daily life through self-control, and v) family and work: between understanding and disbelief. However, whilst our participants often hid their condition and there was some stigma associated with it, they didn’t speak of feeling ashamed. Other studies have highlighted the negative impact that living with chronic headache/chronic migraine can have, emotionally and psychologically, on relationships, careers and finances [18, 19].

Improvement, or perceived improvement, is possible but deterioration also occurs. These fluctuations may in part be the result of life events (e.g. employment issues, family problems etc.) not directly related to their condition. Such events seem to impact on the persons abilities to live with and cope with their headaches. We see that people move in and out of different phases of coping and not coping with their headaches. Although contextual factors make a huge difference in their headache management, our data suggest that the severity and/or frequency of headaches is the main driver of the state of coping with their headaches. Participants reported migraines that were unpredictable and could not be sure how well they would be coping with their headaches at a future time. This is similar to the findings of a qualitative synthesis which highlights headache as a driver of behaviour either directly or indirectly affecting many aspects of life including their relationships often evoking emotional responses of guilt, worry or uncertainty [10]. Indeed, another study looking at developing a strategy to measure outcomes from the patients’ perspectives for use in evaluating preventive treatments for migraine found, that chronic migraine impacts physical functioning, social and leisure activities, and also has emotional impacts. These impacts are experienced during and between migraine attacks and vary considerably day-to-day [20].

A paper from 2022 suggests that management of migraine requires a biopsychological approach integrating non-pharmacological management alongside conventional biomedical treatments [21]. The experience of our participants supports this but also warns of the challenges of finding relief from migraine. The CHESS study was designed to address both the physical and psychosocial aspects of living with a chronic headache disorder but as noted in the introduction no detectable effect was found for the intervention on the primary outcome (health-related quality of life) at 12-months [7, 8]. A recent article taking a closer look at the outcomes of CHESS did find some positive effects on self-efficacy and noted that future work may want to look at other more relevant outcome measures highlighting again the complex nature of this condition and the need to consider all of these complexities [22].

### Strengths and limitations

A particular strength of this paper is that it includes 41 individuals who live with chronic headache, and that from 26 of these we have longitudinal data. This has allowed us to explore the condition in more depth. All of those interviewed had self-reported chronic headache at the start of their involvement in the CHESS study (i.e. 15 or more days per month for at least three months) and it is clear from their stories that they do live with this disabling condition. However, it could also be seen that this is a limiting factor as many of those included may not have ever received a definitive, specialist delivered diagnosis. Nevertheless, we are reporting on the experience of people living with chronic headache disorders; few of whom will ever see a headache specialist.

## Conclusion

“It’s just part of who I am…” is the title of this paper and we suggest that the sentiment expressed within this quotation reflects the impact that living with chronic headache can have. Indeed, this disabling condition is often more than just an aspect of their lives, it is all consuming in its unpredictability and complex in its presentation and management. Our findings imply that helping people who live with chronic headache conditions requires a holistic, whole person, approach encompassing lifestyles, medications, support and societal acceptance of a frequently misunderstood condition.

### Supplementary Information

Supplementary Material 1.

## Data Availability

Much of the data generated or analysed during this study are included in this published article [and its additional information files and published work]. Reasonable requests for the datasets used and/or analysed during the current study can be requested via the corresponding author.
