# Supplementary material for: “It’s just part of who I am…” Living with chronic headache: voices from the CHESS trial, a qualitative study
Source: BMC Neurol. 2024 Aug 2;24:268. doi: 10.1186/s12883-024-03779-w (PMC11295303; doi:10.1186/s12883-024-03779-w)
Supplement: Supplementary file 1 — Supplementary Material 1. [file 12883_2024_3779_MOESM1_ESM.docx]

# Supplementary material.

### Contents

### Interview Schedules, Pages 1 – 4

### Example Pen Portraits, Pages 5 - 21

# INTERVIEW SCHEDULES

### Phase 1, INTERVIEW SCHEDULE

**Introduction**

- Introduce self
- The research team at Warwick Medical School are currently doing some research into headaches and self-management, to consider ways people cope with their pain, what advice, information and actions are helpful or not and what outcomes are important to people with headaches.
- We would like to ask you some questions about your experiences.
- This takes about one hour. An information sheet about the study has already been sent to read. Do you have any questions about the study? Before we continue with the interview are you still happy to participate?
- Go through this and the consent form to ensure the participant is OK about participating, cover the points about audio recording, data protection, confidentiality and anonymity.

**Topic Guide**

**Topic One – Background**

Tell me about yourself (work/home/hobbies).

- **Topic Two – Living with headaches**

Tell me about your headaches

Tell me about how you manage your headaches

Prompts: use/non-use of interventions, timing and impact of interventions; what influences use of interventions

What is most important to you in terms of managing and living with your frequent headaches?

Explore key problems, barriers and mechanisms of benefit.

- **Topic Three – Help and support from others**

What help have your sought from others? How did you find out about it? What form did it take? What was helpful and what was not helpful?

Prompts: For each type of help – why was it helpful/unhelpful? How has it helped?

- **Topic Four – Advice and education**

What advice/education/influences have you come across? Which have you found particularly helpful/which have you found unhelpful and why?

Prompts: For each – why was it helpful/unhelpful? How has it helped?

**Thank the participant for their valuable contribution and explain the next stage in the process of research and how their involvement will contribute.**

### Phase 2 INTERVIEW SCHEDULE (Baseline)

**Introduction**

- *Introduce self*
- You are taking part in the Chronic Headache Education and Self-management Study (CHESS) research run by Warwick Medical School which is evaluating a headache self-management programme to improve the quality of life for people living with chronic headaches.
- We would like to ask you some questions about what it is like to live with frequent headaches and your experiences in the study so far.
- This takes about one hour. An information sheet about the study has already been sent to read. Do you have any questions about the study? *Go through this and the consent form to ensure the participant is OK about participating, cover the points about audio recording, data protection, confidentiality and anonymity.*
- Before we continue with the interview are you still happy to participate?

**Topic Guide**

- **Topic One - Background**

Background information about their life (work, home, family)

- **Topic Two – Living with headaches**

Tell me about living with your headaches

*Prompt if necessary – “Is there something more?”*

*Then prompt for life areas about home, work, social, recreational, hobbies, spiritual*

Tell me about how you manage your headaches

*Prompt if necessary, “You have told me ‘a lot’ quite a bit’ about how you manage your headaches is there something more?”*

Tell me about your medication

*Prompt if necessary, “Tell me more”*

- **Topic Three – Expectations**

Explore what they think will happen in the study

What would they like to happen?

What would life be like if you did not have headaches? *“Is there something else?”*

- **Topic Four – Study recruitment**

Explore reasons for becoming involved, explore contacting protocol and documentation.

Informed consent, process, ease of completion.

Classification interview

- **Topic Five – Completion of study paperwork and electronic diary**

How did they find completing the relevant paperwork including questionnaires and headache diaries (both paper and electronic)?

**Thank the participant for their valuable contribution and explain the next stage in the process of research and how their involvement will contribute. Ask if it is ok to contact them after they have completed their 4 month questionnaire.**

Phase 2 INTERVIEW SCHEDULE (4-month)

**Introduction**

- *Reintroduce self. This is the second interview*. **(note as noted in the methods, to ensure that the sample included enough participants who were exposed to the CHESS intervention, additional participants were recruited at this 4-month timepoint. In these cases, the background information asked in topic one reflected that asked in the baseline schedule (above))**.
- You are taking part in the Chronic Headache Education and Self-management Study (CHESS) research run by Warwick Medical School which is evaluating a headache self-management programme to improve the quality of life for people living with chronic headaches.
- You have already helped by being interviewed at the beginning of the study and have given us valuable information. We would like to ask about how you are now and your experiences of the study since we last met.
- This takes about one hour. Do you have any questions about the study?
- *Remind them about the consent form they have signed (show them a copy). Remind them about audio recording, data protection, confidentiality and anonymity and that their participation is voluntary. Ask them whether they are OK about continuing to participate.*

**Topic Guide**

- **Topic One - Background**

Background information about any changes in their life since the first interview

- **Topic Two – Living with headaches - since the first interview**

Tell me about your headaches

Tell me about how you manage your headaches

Tell me about your medication

- **Topic Three – Completion of study paperwork and electronic diary**

How did they find completing the relevant paperwork including questionnaires and headache diaries? (both paper and electronic)

- **Topic Four –**

**Control**

What did you think about the information given? (*Show relaxation CD and a copy of the letter they were sent)*

Explore what aspects they found useful and why and conversely what wasn’t useful and why.

How would they make it better?

**Intervention**

What did you think about the group intervention?

Explore what aspects they found useful and why and conversely what wasn’t useful and why. Explore tutoring and content. Explore any attendance issues. Enquire about duration, location.

Explore what they thought about the support and its availability.

How would they make it better?

Would they recommend a course to others, or not and why?

- **Topic Six – Exploring change – since the first interview**

Explore any changes in relation to their headaches. What were these attributed to?

**Thank the participant for their valuable contribution and explain the next stage in the process of research and how their involvement will contribute. Ask them if it is alright to contact them again when they have completed their 12 month questionnaire.**

Phase 2 INTERVIEW SCHEDULE (12-months)

**Introduction**

- *Reintroduce self.* *This is the third interview*.
- You are taking part in the Chronic Headache Education and Self-management Study (CHESS) research run by Warwick Medical School which is evaluating a headache self-management programme to improve the quality of life for people living with chronic headaches.
- You have already helped by being interviewed before and have given us valuable information. We would like to ask about how you are now and your experiences since we last met.
- This takes about one hour. Do you have any questions about the study? *Remind them about audio recording, data protection, confidentiality, anonymity and that their participation is voluntary.*
- Before we continue with the interview are you still happy to participate?

**Topic Guide**

**Topic One - Background**

Background information about any changes in their life since the second interview

- **Topic Two – Living with headaches - since the second interview**

Tell me about your headaches

Tell me about how you manage your headaches

Tell me about your medication

- **Topic Three – Completion of study paperwork**

How did they find completing the relevant paperwork? Including questionnaires and headache diaries (*both paper and electronic*)

- **Topic Four – Exploring change – since the second interview**

Explore any changes in relation to their headaches. What were these attributed to?

Explore what happened with any changes identified at the 2nd interview.

- Additional topics arising from previous interviews

**Thank the participant for their valuable contribution and explain the next stage in the process of research and how their involvement will contribute.**

# Examples of actual pen portraits and each of the four categorisations that emerged.

###

Below you will find four pen portraits. The methods used to produce these is included in the paper. In brief, these examples are all from a single timepoint (baseline).

Each portrait is presented under four headings.

- Different headaches (their headaches and their journey)
- Quality of life (e.g. impact on life)
- Managing their headaches (e.g. coping etc.)
- Medication (relationship with their medications and their health professionals)

The data that populates these is a mixture of researcher notes/interpretation/discussions and actual quotations from the interviewees (as exemplars). At times you will find that we leave in the questions asked of the interviewees to provide context for the responses.

Each of the examples represents one of the four categorisations developed in this work. These are.

- I = I will not let headaches rule my life,
- II = Headaches rule my life,
- III = Headaches out of control - something needs to change,
- IV = Headaches controlled - not ruling my life

As explained in the paper you will see that all these people have complex lives and their relationship with headache is also complex. The predominate category was carefully considered by the research team as detailed in the methods. In all cases there are elements of other categories which are evident in the data. The conceptual model presented in the paper helps to illustrate these overlaps.

### Participant 19, Baseline, categorised as: I, “I will not let headaches rule my life”.

Even though they have regular headaches they do not let them stop what they’re doing, uses distraction and gets on with their life. Feels they would experience their headaches more if they focussed on them or talked about them.

## Different Headaches

Demarcates headaches into those which they can ignore with or without medication and those which they have to stop (which occur less often).

*I can have anything from 8 to 12 a month it’s very rarely under 8 and it’s on a rare occasion it can go up to 13 or 14 but that’s quite rare now so I have between 8 and 12 a month now… and they are not… they vary… mmm… in degrees you know sometimes they can go up and down and… err… and other times I can manage to go on with the migraines… 80% of my migraines are at night… mmm… and 50% of those will start in the late evening an 40% will start in the middle of the night… they wake me up in the night… …no in the middle of the night… 2 or 3 o clock I’ll suddenly wake up with a migraine… migraines generally last between 8 and 10 hours although if it’s a really bad one I can have them from 2 or 3 days you know not… not bad… one day will be really bad and then I’ll out for 2 or 3 days and then I might have a migraines during that time…*

*The bad one I will have to stop and sit quietly or come in here… mmm… close the curtains and just sit it out really… rest it out… mmm… but those are really painful and… mmm… I mean they last for… the severe pain lasts for about three hours then it tends to go off and I’ll go and sit read or watch tele quietly or something like that I try and find something to take me mind off it as soon as I can but when it’s really bad it’s quiet and the curtains closed and I mostly in bed… basically I’m in bed when that happens… I’m already in bed in the dark so…*

## Quality of Life

Tries not to let it control their lives. Headaches can affect their morning routine and occasionally social or work activities, but they are determined not to let it control their life. They maintain a positive outlook and try to avoid dwelling on the pain to prevent it from affecting their mood.

*Well they will if… mmm… err… I have bad one or… because they… the majority happen at night it means that basically my day… it takes me more time to come round in the morning… … so it might be 11 o clock before I come around and get out into the garden so that I suppose whereas before I would’ve… mmm… got up earlier and got out there and if I’m doing certain jobs… mmm… like a hammer drill I have to be careful because that will certainly give me… err… if I do too much of it… it will give me a migraine there’s no two ways about that… the sun will give me a migraine if I sit in the sun…*

*…occasionally it will… mmm… disturb the social things I do or work… but it has to be really, really bad for me to… to do that and I can’t say it hasn’t happened it’s probably happened on quite a few occasions but… if you dwell on the migraines well for me if you think about them and that they will only get worse because you are just thinking of the pain and you want it to go away but I find if you just carry on… err… you… it’s difficult to start with because… but it’s something I’ve got used to I suppose… mmm… more than anything so… mmm… it doesn’t disturb my life… err… that much but that’s because I won’t let it…*

*… emotionally… it very rarely it gets me down it’s… I can’t say it hasn’t and it doesn’t… mmm… but that’s when if you sit and worry about your migraine that’s when you do start to get depressed I feel… I think trying to ignore it will stop you going down I mean everybody’s different so you know it’s… err… you know that’s how it works for me so I try not to get too… I am not saying it doesn’t get me down ‘cause it does especially when I have three on the trot in the night and you’ve hardly had any sleep so it’s really the migraines getting me down or the lack of sleep it’s probably a bit of the combination of the two and that can make you a bit low… mmm… but I’m very fortunate I’m… I don’t… mmm… I have a positive outlook on life I feel the glass half full rather than half empty… and I just refuse to you know it’s not going to win you know it is a battle between me and the migraines…*

*… my wife will organise our social calendar as if I haven’t got migraines and this is what I want her to do and it’s very rarely that I will say, ‘ no I can’t go out’ … …I might be tired and I might be drowsy and I might be you know a bit heady… well usually am but I can… I can function so I will go out because… it can easily wreck my life and wreck me wife’s life as well and I’m just not gonna let that …*

## Managing their Headaches

Avoids known triggers and tries not to let the headaches rule their life. When they sense a headache coming on, they might take a couple of tablets but continue with their planned activities. They have an impressive degree of control prioritising social interactions and overriding the pain to engage with those around them.

*I obviously try and avoid flashing situations lights… … I’m careful how I do the DIY so not too much bending as bending down can start it sometimes… I’ve just finished painting the garage doors and the sun’s been on me back and the back of me … … the sun could cause the migraine… or it’s… so I think it would just… there is no… but obviously I try and avoid things like that… I don’t eat chocolate and I don’t eat… mmm… err… cheese and cream…*

*I never know if it’s going to be bad or what… no it… will only change what I’ll do when I’ve got the bad one not when I think it’s coming on… if I think about when a headaches coming on I might take a couple of tablets but I’ll still continue to do what I intended to do or go where I intended to go… err… and it would have to get really bad for me to stop at home… I might be sitting quietly in the pub or whatever and it will probably be more painful if I’ve got it because of the noise and the atmosphere but I just don’t… I would never go out you know it’s… you would just never do anything really so I’m just… and again people talk to you and you tend to override it if you like the pain… and I make the effort to make conversation…*

## Medication

This participant has tried many different medications in the past. Now uses ibuprofen and has regular Botox which they feel has helped to decrease the frequency of their headaches and encouraging for them to find a treatment that works effectively without significant side effects. They monitored NICE guidelines, waited until Botox was available on NHS in their area and then went through their doctor to access it.

*… I just take normal migraine tablets [ibuprofen lysine]… err… for it I have tried a lot of tablets… Migraleve and all that but a lot of them contain Codeine and I don’t particularly get on with Codeine so… so I just have… err… normal headache tablets unless they are really… sometimes I’ll feel one coming… if I’m… if I’m going out and I feel one’s coming on I will have a couple of tablets… err… but where if I wasn’t going out I wouldn’t have had them!*

*… when I first had the Botox it was quite dramatic really because I had the Botox and within two or three weeks my… my migraines were reducing to about 10 a month rather than 15 a month so it really cut them down to (inaudible) so and 3 migraines a month less which was probably the (inaudible) it could be up to 5 migraines a month less is fantastic it really is because you have three to five extra days a month that are pain free so it’s actually brilliant there’s no two ways about it and I think because I’m on this… err… system of having it regular it’s the same time every three months it’s probably improving more because it gets into your system and it just… so I… I’m fairly convinced that if I didn’t have it that within probably three months I would be back up to 15 a month…*

*…… it [Botox] has made a fantastic difference to me there’s no two ways about it and… err… err… and because of… I’ve been for years and years and years and nothing had worked and I’ve constantly been having these migraines for so long to find something that actually worked that… there are really no side effects like in the medication…*

### Interviewee 17, baseline, categorised as: II, “Headaches rule my life”.

## Different headaches

Used to get more severe migraines now one a month 'bad'. But generally, has a headache 3 times a week and a niggle most of the time.

*Mmm… I had one last week probably… I probably get one a month now and it’s not really bad but I think that’s a build-up of being tired.*

*Yeah, headaches … mmm… sometimes I feel like I’ve always got a headache I mean even now I am sitting here I’ve got a throbbing here… not bad but I can feel it here… it won’t come to anything I know that but it’s like my head never feels clear… I never feel fantastic there’s always something niggling away… mmm… but not bad headaches but headaches that affect your day I probably get three a week on average… last week I had one… I think I had one Monday, Tuesday and Wednesday… ‘cause even if I get a bad headache it will still go on for two days at least……and then it probably hasn’t finished with me then it will come back… I may not have it in the morning but it may come back later in the day sometimes… sometime… so I always feel like there’s something there.*

## Managing their headaches

Mostly managed by medication but uses walks in fresh air if not too bad or lying in a dark room if really severe. Tried acupuncture in past which helped. Drinking water (doesn't help) Sitting quietly does help a little. Finds they cannot skip meals and has to eat regularly. Has used diaries in the past and notes headaches on calendar for the phone App. on CHESS. Sensitivity to Light and Noise:

Photophobia (sensitivity to light) affects them during headaches, making bright environments and noise unbearable. Even sorting their disabled adult son can be challenging due to the sensory overload.

*...bad migraine which some of them are complete agony you know absolute excruciating… mmm… those Triptans won’t touch it so it’s just this… it’s just a case of going to bed and just you know… that’s it for the day… wipe the day out and forget about the day… you see what I mean?*

*… I’ve tried drinking loads of water… somebody was telling me at work once that if her daughter gets a headache come on she has a good drink of water and it goes off… so I’ve tried that……that’s not worked… mmm… really sitting down and being quiet but I rarely get time for that… mmm… especially… well… if it’s in the day time and my son’s at school or college I’ll just try and sit be quiet you know that helps you feel a bit better but it doesn’t actually take it away but you actually feel better because you are sitting quiet… not moving your head… ‘cause when you move your head it’s like this throbbing so… so when I’m sorting my son out and I’ve got you know… you are changing him and everything and moving my head it’s just… it’s just whoo, whoo, whoo it’s all in the back of my eye… mmm… so sitting quiet going to bed and draw the curtains… just lying on the bed with the curtains drawn… dark room.*

*‘cause it’s… it’s the light affects it… light and noise… what is it called… is it photophobia or something where the light affects ya… yeah… ‘cause everything just seems brighter when I’ve got a headache everything seems brighter… everything feels louder… mmm… that’s why sorting my son out is horrendous because he… ‘cause he shouts… he’s got his noisy toys and it’s just like… I’ve even used earplugs ‘cause it just like… I feel like my heads going to explode!*

*… I had Acupuncture for years… when did I start going… probably something like 1998/1999 I started going for Acupuncture and I’ve only stopped going the last sort of five years… four years, five years something so that helped… it… it reduced the frequency and the intensity but it didn’t cure it… mmm… but I felt like I was doing something positive you know to try and get rid of it and it helped and to be honest you’d try anything…*

*but if I’ve got a bit of a headache… not a bit of one… if I’ve got quite… a… headache and feel I can go for a walk then that’s what I do but if I hadn’t got the dog I wouldn’t go… there’s no way I’d go for a walk down the road on my own so that helps he gets me out sometimes I feel better for it… sometimes it’s made it feel worse but… so…*

*… that’s another thing that I forgot to mention I have to eat regularly I can’t skip meals……because whether it’s something to do with blood sugar levels dropping… mmm… if I don’t eat properly… mmm… I’ll get a migraine so I have to… I can’t skip a meal so if we are out all day and I’m hungry we HAVE to eat… I have to find somewhere to eat… mmm… I can’t miss breakfast under no circumstances ‘cause I… that’ll really make me poorly…*

Using a headache diary*. … it makes you recognise… mmm… the frequency it makes you recognise… ‘cause anything I’ve read about migraines and I’ve read a lot… I’ve trawled the internet… I’ve read loads… they all say keep a diary because it makes you recognise what’s triggering them… when you get them… mmm…if it’s part of your cycle… mmm… so it makes you… it makes… it hi-lights some factors and it probably helps you because you can tackle those things then… yeah.*

## Medication

Uses propanolol as preventative and cocodamol as first choice when headache starts or triptan if definitely a migraine. Triptans sometimes work well sometimes don't work especially if been sick. Will take a cocodamol if they have to do anything and they have any kind of 'niggle'. Fearful of coming off propanolol in case they went back to having the bad headaches. Faces dilemma of choosing between weight gain (due to Pizotifen) and headache relief which is a tough decision, which if often made based on their priorities.

*… lots of tablets… I just… I take Cocodamol… Paracetamol doesn’t even come anywhere near… mmm… so I take Cocodamol… mmm… sometimes I’ll wait and see how it’s progressing and I think oh do I need to intervene with medicines or can I just… can I put up with it… which a lot of the time I just put up with it… mmm… other times… say if I’ve got something on or I’m going out or I’m going shopping or something or that’s about it… go shopping I’ll take some tablets if I feel I’ve got the slightest little niggle I’ll take some tablets and I’ve thought I’ll stop it… I’ll stop it in its tracks…it doesn’t always work…*

*… sometimes I feel like I live on Cocodamol… you are not supposed to take it you know… mmm… I think it’s more than five consecutive days but sometimes I just feel like I live on it…... so I’ve got… I take… err… what’s it called… Propanolol……I take that daily… that helps…*

*… they’ve all helped… they’ve all helped in a way I mean I still take the Propanolol and I wouldn’t like to come off it ‘cause I don’t want to get back to that stage I was at… mmm… the Triptans they are good if it’s like a sort of… if it’s not going to be a mega bad migraine they are good… they will sort of… I can get rid of it within an hour… last week Wednesday morning I got up with a bad head but you lie there in bed and you think… oh I can’t move me head but I’ve got to get up to get a tablet so you lie there and you think I’ve gotta get up… so you want to stay in bed ‘cause your head hurts but then you’ve got to get up to get this tablet which is gonna take it off so I took one of those last week I’ve got a busy day on the Wednesday so I took one of those… mmm… after an hour it’d gone.*

## QOL

They started off by saying that ‘headaches ruled their life’. Not at work at present due to being main carer for their disabled son. Headaches affected work when they were at work. Headaches restrict their social life and hobbies although these are also restricted by their carer circumstances. Sometimes they find it difficult to manage their carer role when they have headaches. Emotionally their headaches ‘get them down’. Whilst there is an underlying will to try different ways of managing their headaches personal circumstances and homelife are making this very difficult.

*… it just controls everything it… it controls where I go… mmm… I felt unreliable at work you know… mmm… I’d have to cancel appointments there’s times I’ve had a hair appointment and I’ve had to ring them up and cancel it sort of an hour before I’m due there which you know… it’s just completely controlled my life… mmm… hated… hate traveling because I know I’ll get a headache… it never… it never stays as just the headache it always progresses and gets worse and worse and then I end up with a migraine.*

*… especially with my (young adult) son it affects it a lot because he’s disabled… he can’t talk… mmm… so he shouts a lot… when you’ve got a splitting headache… a splitting headache and a disabled child… don’t go together because I’ve still got to change his pad I’ve still got to do everything he needs… I’ve still got to cook him something… (I can’t say) look I’m going to bed get yourself something to eat… I can’t with [son] I’ve still got to make him something to eat… still got to change his pad… mmm… I actually have carers come in now because he’s an adult I have carers come in the morning and in the evening to get him up ready and that in the morning and get him bathed and that at home but that’s only been running for about 18 months so I’d still have to bath him of an evening… mmm… you know he’s a strong lad he… you know it’s not like bathing a baby it’s you know… mmm… so get him something to eat I have to feed him… I have to put him to bed… change him again put him to bed and you are doing all this with this horrendous pain in your head you know…*

*[son] is pretty much a full time job… mmm… I can’t… I can’t go out of an evening… obviously because I can’t leave him on his own… mmm… I’m just… I’m trying to get respite at the minute so I’m hoping to get back on my hobbies… mmm… there is one hobby that I love and that’s me photography and I’m thinking of joining a group and they go away for days out and that and I thought oh no I wouldn’t be able to do the days out ‘cause what if I’ve got a headache when I get up ‘cause you are going out for the day and I just used to think, ‘No! How can you be happy and sociable when you’ve got a splitting head!’ so… I quite… it has made me quite reclusive if I can go that far… I mean I had the dog last year and mainly I had him to make myself go out you know and even if I’ve got a headache I go out with him and it sort of blows the cobwebs away and you feel a bit better for having a bit of exercise… yeah but no… no social life… no hobbies!*

### Interviewee 15, Baseline, categorised as: III, “Headaches out of control - something needs to change”.

## Different Headaches

They are experiencing permanent headaches since having a brain haemorrhage. Sometimes, the sensation is more of a thumping without intense pain other times, they are having actual pain, often on the left side, similar to a normal migraine the sharp pain they describe as akin to brain freeze from consuming cold treats.

There are some indications that this person has a degree of control over their headaches and its impacts but as one reviews the evidence around the management of their headaches, their QoL and their relationship with medications it becomes clear that things do need to change.

*Mmm… I’ve had headaches now for just under five years… well I mean like permanent headaches that started after a brain haemorrhage… mmm… they are there all the time but not always that painful because sometimes it’s more of a thumping sensation rather than actual pain and then sometimes there’s actual pain as well so sometimes I feel I’ve got a headache but actually it’s… it’s like headache without the pain but then other times I have a headache as well… …things that make them worse is lights, driving using white paper……it’s all to do with reflectiveness so… mmm… it’s gets reflected off white paper so if somebody handed me a bit of white paper and the light reflects off it I get like a buzzing sensation in my head it’s like an electric shock…*

***…****Normal pain is when… well actually it’s normally behind… it’s normally focussed on the left-hand side of my head like that like behind my eye… …that’s what I consider as treatable pain if you like!*

***…****The normal kind of thumping sensation I often feel quite sick with it I can take anti-nausea pills they help but actual… for pain it doesn’t… it doesn’t get rid of that kind of thumping sensation so… so there’s no point in taking more medication than you need if it’s not doing anything!*

## QOL

All aspects of quality of life are severely affected. Some adaptations to their activities, such as attending Pilates and positioning themselves away from patterns and bright lights. There is disappointment and levels of depression at having to give up hobbies/activities. Finds they have to go to bed for an hour or two when you get home and are not often able to do everyday tasks like cooking a meal and playing games with the children. Has days when they feel that headache is out of control and worries about being able to work and other aspects of life. Socially it has become very difficult to engage with anything. Has some clear, major, anxiety/depression issues which seem to be ongoing with support from professionals, family and church.

*Yeah I do go to Pilates… well I suppose it’s not socially but it’s exercise…*

*…yeah so I do that cause I’ve not got a very good back so that keeps me mobile and I wear my dark glasses I have my sunglasses and I position myself not directly underneath the lights so yeah and I go near the front because I can’t stand stripes and patterns… I have that… you know when people get sick with patterns and it makes me sick with patterns so I have at the past been at the back with someone who’s come in late with strips on her and I’ve thought oh no I cannot… I cannot stand watching this so I position myself carefully according to… the same at church I sit in a certain place at church because I know that if anyone comes in I won’t… I am near the front so nobody can go in front of me with a stripy top on… you actually do it automatically now… I automatically go to the darkest bit of the room without even thinking about it!*

*… I did get really depressed ‘cause I had to give up… I tried to go back to hobbies like things like karate and running and stuff like that and then discovered that I couldn’t do it… the thing is when I came out of hospital I felt so much better as you do… I felt that I was gonna be fine and I think the surgeon’s idea of fine and my idea of fine ‘Oh it went well’ that’s what he said. ‘ it went well’… I think his idea of ‘it went well’ was him saying you are still alive and you are ok… my idea of it went, well you would be 100% fine but then these things manifested so I got really quite depressed…*

*Mmm… I nearly always go to bed when I get home for an hour or two I very rarely don’t… I don’t… I don’t always go to sleep but I always lie with my eyes shut I quite often put the tele on but I don’t really watch tele ‘cause I don’t do screens… …so I quite like… like you know game… not game shows… quiz shows because I can listen to them when they read out the … … I don’t need to actually look at it which is quite good… so I lie there and listen to that and then that’s about 6 o clock after that my husband makes dinner during the week… I don’t cook during the week the fact that I’ve got a white shiny kitchen doesn’t help… we had it put in the summer before I had the brain haemorrhage so it was quite new, so he does the cooking during the week…*

*…I mean after a couple of hours I am fine for the evening… I just need that break that’s because I usually drive I always need a rest after I’ve driven ‘cause obviously not doing reflections and looking in mirrors and all the things my head gets sore almost straightaway when I drive a car.*

*I usually sit with my eyes shut most of the time especially if it’s sunny and I’ve taken to sitting in the back when the kids are in the car and they sit in the front because we’ve got all the dark… blacked out windows so I sit in the back.*

*… I don’t really watch much tele… I watch a bit with the kids but I rarely see a film because my head I just give up ‘cause of the light and playing board games and things I find that really hard… cards and things I don’t really do that anymore so yeah!*

*it’s just sometimes my headaches… it’s almost out of control… it makes me really depressed and it’s not because I am thinking ‘Oh my head hurts’ it’s almost like a really oppressive feeling in my head and it makes me really depressed and sometimes there’s no real reason for it… it’s not like something bad’s happened or I’ve had a bad day… I’ll just wake up and that’s how I’ll be and my husband will say you are not right… you are not right today!*

*Well I think the anxiety is caused partly by having headaches all the time… maybe not my headache itself but the effect that my headache has on not being able to do my job properly… not being able to do the (inaudible) letting people down and all the things that go with not being a 100% so my anxiety comes from the fact that… that you know sometimes I just cannot do marking and stuff because my head… I just feel too sick and then there’s the worry about or what if I’m like this tomorrow or I still can’t do it and then what you know that kind of anxiety but you… because other people… what I used to do is if I needed to do marking I could stay up all night and do it now I haven’t got that option so I have to plan really carefully that if that doesn’t work ‘cause I’m not well enough to do it one day then I’ve got the kind of I don’t know if I’ll be able to do it now it’s a sort of worry… so it’s that anxiety coming from the headache …*

*Socially… mmm… I don’t do socially now much anymore… I try… I used to not go to things because it was like… I’d used to be very much an all or nothing person so I’d think I’m go… if I go I’ll feel sick because if it’s somebody’s house it’s their lighting quite often I have to leave it’s different types of lighting or if there’s a lot of people talking I really do noise either so if there’s people talking I find that… I think my brain’s just tired and I just find it too much so… mmm… if I do go I tend to say I might come for a bit so it’s like I went to my friends 50th and it was an all day party so I just went for the afternoon and didn’t do the evening luckily I didn’t have people talking and I do a church group once a month but I very rarely make it because I nearly always have a mi… a very bad headache by Thursday… Thursday evening usually having done three days at work and Thursday is quite a busy day at work I usually just… I intend to go but I just think my head hurts too much and I can’t go… I’ve been and been completely miserable and people have said are you alright and yeah I’ve just sat there because I can’t read the book… can’t read my bible and sometimes in the week we go to different people’s houses to stay in the group and if the lighting isn’t very good and I can’t stand the lighting and I just think what am I doing here so I do not do a lot… I am quite careful what social things I do.*

*… I’ve been a Christian for quite a while but I was kinda of… I kinda of took five years out almost for when my Mum passed away… I just found church too difficult but then when I was in hospital I just felt that I’d be ok I just felt that being spiritual that God was with me and that’s… somehow it would work out for the best and not just about all the church gave my family even though we’d stopped going to church…the minister was a friend of ours who used to come around with meals and they had a meal rota and they’d pick the girls up ‘cause we had the kids to look after and I started going back to church and I really don’t think I could… I would cope nearly as well if I wasn’t… if I didn’t have the support of the church and going back to my (inaudible)… I don’t see them that often but… mmm… they… they’ve got a list of phone numbers for me to call if I’m depressed and I’m feeling like doing something stupid ‘cause what I used to do was get in my car and drive up the motorway to de-stress so I’ve got a list of people to phone now so if nobody’s in on the first one I can phone somebody else so they’ve put all that together for me so yeah they support me… the church supports me and I do feel like that it has become a bigger part of my life.*

## Managing their Headaches

Has a reasonable relationship with GP who also seems concerned about their mental health and well-being. Wants to work but headaches do impact this. When they do work has to manage environments, noise etc. and takes rests when they can. No clear plan for the management of the headaches, they are hugely disruptive with currently littled respite.

*…so I got really quite depressed and worried about being able to keep my job because actually I had only just started my job when it happened…*

*…Yeah… I’ve had a change of GPs because my GP left but my new GP he actually contacted me first of all he did say for a while he thought I was really not in a good place so to go and see him every couple of weeks just to chat which is good because I wasn’t getting any counselling ‘cause he’d referred me and it didn’t happen but he would see me to see that I was ok and see how I was getting on I mean he put other things in place because I’d had a bad back so he put into place to try and sort that out… so sort of focus on the things that we can fix as well just to be a bit more positive.*

*…and I can ask and I can say to him look I am really not right I want to go to work because I don’t want to let the students down but I can’t see 20 students with all their problems at once so he wrote me a letter to say… a fit note to say, small groups only and that sort of thing so he’s good and he knows that I want to be still at work even though I find it hard sometimes so he’s supportive that way as well…… I work four days a week even though I work a point six so it’s like four days instead of three because I can’t teach too many lessons in a row so my limit is really to teach three classes so I have a lesson… I have… on my time table I have four classes on a Tuesday and one of them has been moved to another day so that holds me to three days… three lessons at the most and quite often in my lunch hour I go and lie down in the medical room so if nobody can find me that’s where I am… I also do have… I don’t really have time off work as in like whole days off work there have been times when I’ve not managed to go in first thing in the morning ‘cause I’ve had a migraine or I’ve had to go home early because I’ve had a migraine…*

*…I’ve not managed to last … and when I had four classes in a row… err… I really quite often missed the last lesson… that really… I couldn’t really cope… when I get quite anxious about it ‘cause I get quite like… I think if they ask me one more question I’m just going to be sick and sometimes I’m sick I quite often run to the loo to be sick… (inaudible) ‘yeah you’ve been sick in the corner’ but that’s just the way it is… my work is understanding so it is quite well planned my time table.*

*… So now it’s quite well planned out like I teach normally in the morning from about 9 and I finish by about 2.30 so I’m not doing to the end of the day.*

*…it’s all to do with reflectiveness so… mmm… it’s gets reflected off white paper so if somebody handed me a bit of white paper and the light reflects off it I get like a buzzing sensation in my head it’s like an electric shock…*

***…****so in my lessons at my college we all use blue paper… even the white boards that we use mini boards are blue……so I’m a blue person classroom… that makes a huge difference if I try and mark a white paper I can’t mark it for very long… I just get headaches and I start being sick it actually makes me vomit. …Well we all use blue paper in my class room and things like… err… my PowerPoint slides have got a blue… I’ve put them on a blue background… everything… it makes it easier for students to find their work because for me it’s blue! (laughs)*

*Well we all use blue paper in my class room and things like… err… my PowerPoint slides have got a blue… I’ve put them on a blue background… everything… it makes it easier for students to find their work because for me it’s blue! (laughs)*

***…****How did I find out that blue would be a good idea… mmm… I went to an ophthalmologist who suggested I saw an optometrist with the blue overlay thing and I found out what the best colour was. I’ve since been tested more thoroughly for possibly (inaudible) and they’ve come up with a better combination so a combination of the different blues you know like optimise it so that’s how they’ve come up with it.*

*I started going back to church and I really don’t think I could… I would cope nearly as well if I wasn’t… if I didn’t have the support of the church and going back to my (inaudible)… I don’t see them that often but… mmm… they… they’ve got a list of phone numbers for me to call if I’m depressed and I’m feeling like doing something stupid ‘cause what I used to do was get in my car and drive up the motorway to de-stress so I’ve got a list of people to phone now so if nobody’s in on the first one I can phone somebody else so they’ve put all that together for me so yeah they support me… the church supports me and I do feel like that it has become a bigger part of my life.*

*… what I used to do is if I needed to do marking I could stay up all night and do it now I haven’t got that option so I have to plan really carefully that if that doesn’t work ‘cause I’m not well enough to do it one day then I’ve got the kind of I don’t know if I’ll be able to do it now it’s a sort of worry…*

*…my headaches are more related to what I’ve been doing or what I have to do you know how much time I’ve spent on the computer, how much marking I’ve done, have I driven my car which is why I have them every day because these things are triggers that I can’t avoid even watching tele you know if I’ve got a headache I just have to lie in a room with my eyes shut.*

***…****I’ve got blinds in my lab I’ve got special anti-reflective foil on my windows so it… I’ve come pretty far in managing it really!*

*… I listen to a lot of music I would be quite happy to spend a lot of money on music because I even sleep with music on when my heads bothering me… …it’s my distraction… I use music at work a lot to distract me from my headaches if my head’s driving me mad at work and I can’t use something because it’s going to make me tired…*

## Medication

Medication Approach: They seem to differentiate between the thumping sensation (which doesn’t respond well to painkillers) and actual pain. Sumatriptan helps fall asleep providing a break from the discomfort. Paracetamol and Codeine or Paracetamol and Naproxen are your go-to options for treating pain. They have tried various preventative treatments, including beta blockers, Amitriptyline, and Botox injections. They have undergone Botox injections twice, with some improvement after the first round but not significant reduction in headaches. Propranolol, primarily used for anxiety, also serves as a preventative for migraines. Generally, a feeling that medications are not managing their condition well. Personal choices also seem to suggest that they strategically decide which medications to take based on nausea discomfort and specific situations. For this person their relationship with medications (what works and what doesn’t) and all other aspects of their lives is very interlinked and on many levels things need to change.

*Mmm… yes because the thumping sensation doesn’t go away no matter what. So I’m stuck with that taking painkillers doesn’t really have a lot of effect unless I take something like Sumatriptan which makes me fall asleep so I get a break if I take something that makes me fall asleep… whereas when I’ve got actual pain then painkillers do work… might still have the sensation of thumping but I get rid of the pain part so I take Paracetamol and Codeine or Paracetamol and Naproxen and it will help so there’s no point in taking it for the thumping sensation because it just doesn’t work… I try and keep the painkillers for normal pain.*

*The normal kind of thumping sensation I often feel quite sick with it I can take anti-nausea pills they help but actual… for pain it doesn’t… it doesn’t get rid of that kind of thumping sensation so… so there’s no point in taking more medication than you need if it’s not doing anything!*

*… I’ve tried just about every single combination of preventative treatments there is…*

***…****Well you can take preventatives to stop help prevent headaches like a beta blockers and Amitriptyline… I’ve also had Botox injections… I’ve also been in hospital for five days having infusions which they give you in an intravenous drip three times a day for five days and it’s supposed to help and after two months you are supposed to have a follow up appointment to see if they’ve worked but I still haven’t had mine seven months later I have been in touch with them and I have now got an appointment in September but I was supposed to have had it in March so that’s the NHS for you… but none of them have worked but I do take Propranolol which is used for my headaches but I primarily take it for anxiety but it’s also a preventative for migraines and I also take an anti-depressant I have changed to a different anti-depressant than I was on before ‘cause the one I am on is supposed to be better for my migraines as well as being an anti-depressant so I do take medications which possibly help slightly they don’t obviously help greatly but they… I think it would be even worse without them.*

***…****Venlafaxine and I take Propranolol.*

*… the Propranolol was to help prevent migraines it never really worked for me…*

*…but then I was prescribed Propranolol for anxiety……so it does maybe work a bit for both but it wasn’t very successful as a migraine treatment……which is why I still have headaches… but I get… I was actually shaking quite a lot with anxiety but just with this thumping in my head it’s a bit like when you hurt yourself and you go oh I was doing it all the time. So Propranolol helps that anxiety part of… of stopping headaches.*

*So I had Botox twice you have to have a minimum of 15 headaches a month to have it… mmm… I had it at the hospital… hospital up here… err… I had 30 injections in my head and neck… after the first lot I felt there was a slight improvement but it didn’t reduce the number of headaches and it’s only licensed for reducing the number of headaches and then I had it again and I didn’t think it did anything the second time so it didn’t really work and the other thing is…*

*Right so… mmm… so the ones that I… the things I take as and when are anti sickness tablets… err… Paracetamol and Codeine and Paracetamol and Naproxen. I decide what to take and when it’s quite… quite interesting because I have quite specific reasons for taking what I take… mmm… if I feel nauseous in the morning and I’m going I’ll take my anti-sickness tablets ‘cause I don’t want to be sick when I’m out… or if I feel sick during a lesson I might go and take them I might pop out… I might say I’m just popping out and I’ll take them then so I don’t take… I can take up to six a day so I don’t necessarily take all six a day just take as and when… mmm… if I have a headache and it doesn’t matter if I’m tired I’d take Paracetamol and Codeine…*

*…so I am not going to drive the car… codeine makes you quite drowsy you see…*

*…so I wouldn’t take that… so if I’ve got to drive to work whatever I wouldn’t take Paracetamol and Codeine…*

*…so if I did have a headache I would take Paracetamol and Naproxen…*

*…because that doesn’t make you too tired…*

*…so I’ve got to be careful about what I take… whereas if I’ve got a headache and I want to go and have a lie down I would limit it to some Codeine ‘cause then I would just… it would help me sleep…*

*…so I’m thinking about… and also if my back’s bad I take Naproxen because it’s… mmm… good for inflammation in your back….*

*…so that’s kind of the reasons of why I take what it’s more because what I have got to do so I wouldn’t take Codeine if I had to teach a class because I would just be so tired… so I’d have to… but I find if I… if I kind of need a rest then Codeine is a better one to take…*

*Right so Sumatriptan I take when my headaches are absolutely killing me… I feel sick I am not going to be able to do anything so say I know I’m not going to be able to go to work ‘cause I’m probably feeling that ill I would take Sumatriptan because I could take Sumatriptan… I would never take Sumatriptan if I was going to work as it would make me too tired… mmm… if my headaches that bad that I need to take Sumatriptan I’m not going to be able to work… so Sumatriptan is my… head’s driving me mad… can’t bear to look at anything… you know if I can’t bear to look at anything it’s that kind of pain that I just need to lie and shut my eyes I would take Sumatriptan then!*

***…****Sumatriptan is the best one……yes so Sumatriptan always makes me fall asleep for about two hours ok so absolutely no good if… but I do wake up after two or three hours where I feel ok and it’s the only thing which does work… it doesn’t… it’s funny it kind of stops suddenly and I suddenly feel really rubbish again probably because I’m doing too much because I’m feeling better but I have a few hours where I feel actually… I feel ok but you can only take Sumatriptan up to 10 times a month and the only thing is it does completely wipe me out for the first couple of hours after I’ve taken it…… I do have a day about once a week when I’m in bed pretty much all day or at least all afternoon at least a good part of the day I just… I would take it then… I would have to take it then (inaudible)… but it does give me a few hours when I think phew I feel better now!*

### Interviewee 06, Baseline, categorised as: IV, “Headaches controlled - not ruling my life”.

## Different Headaches

Makes the distinction between normal headaches and migraine. Migraines come with additional symptoms like temperature fluctuations, eye strain, light sensitivity, and noise sensitivity. The pain associated with migraines feels extended, like going 12 rounds in the boxing ring. Their most severe migraines involve blurry vision, pressure behind the eyes, throbbing, and grogginess. Sweating is common during migraines, and they sometimes feel nauseous without actually vomiting.

*… but it can be just a normal headache or migraine those are totally different it does kind of get frustrating when people go, ‘oh I’ve got a migraine’ ‘Oh have ya?… have you really got one?… because you know you wouldn’t be able to do what you are doing right now if you have…’*

*If I get a headache or migraines they are totally different.*

*Int: Ok tell me the difference then?*

*Res: Mmm… to me a headache is kind of more slight pain it’s more like just there and you think ‘oh me head hurts’ kind of like a bit like a hangover you wake up and feel a bit groggy feel a become like you’ve been with Mike Tyson a little bit you feel a little bit knocked about and slight pain. Whereas a migraine it affects more of ya like temperature wise how you feel cold, sweat… heat feel really hot, feel really cold, feel clammy… mmm… because sometimes I’ve sat there and I was just sweating and it’s like and everyone else is just around me just normal temperature and I feel like I’m sweating like anything and… mmm… eyestrain… eye pain… light sensitivity to lights, noise sensitivity and stuff like that and it feels like the pain with a migraine is like extended. So like you’ve gone 12 rounds with Mike Tyson rather than just the one punch you know to an extent… So that’s what I kind of use… ‘cause sometimes the reason you’ve got a headache it’s well because I’ve got a bit of a bad headache but then it’s not like that so it’s a bit of the two!*

*The range of it naturally can be anything from over an hour and half to I can carrying on for about three days the longest… with the three day one I just kept going I felt… nothing helped…*

*Res: No I was just thinking about the pain of it can be different obviously!*

*Int: So can you describe the different……?*

*Res: The natural… my severest migraine literally (inaudible) just there from the severity of it like my eyes go sort of blurry… I can still see and still function and still carry on and still see things but things are a bit more distorted……from that it feels like me eyes feel like they are under pressure like they are pushing on the back a bit kind of like my brain’s been expanded. There you go, that sort of expression……I feel like my brain’s just expanding and my head’s just like throbbing. Not just in one place as I say it’s more round my… towards the back of my left side, that’s more the severity of where it is but all around it still hurts… obviously I still feel the grogginess… it’s more like a hangover from a migraine you know… mmm… I do feel sick but I’ve never really been sick from them… I just feel like something’s in my throat, that sort of sickie sort of feeling… mmm… sweating always sweating if I’ve got a migraine… mmm… That’s kind of about it the best to describe them… I think in my own sort of way it’s kind of like yeah my eyes feel a bit pressurised and I’ve got a… my head’s thumping a bit but it’s not like… as it is I go from one round with Mike Tyson to twelve…*

## QOL

## Despite migraines, their QOL remains largely unaffected. They have continued activities like visiting Legoland or Drayton Manor even with migraines.

*Int: So we were talking about the effects on work and what about the effects of your headaches on home life?*

*Res: There is none I’ve been in Legoland or Drayton Manor or whatever else with a migraine (inaudible) and I’ve just carried on. Whether it’s sitting there with the kids or whatever else… if my partner’s there it’s a bit easier I can just sit down but I can still sort of play even if we’ve been out and about I’ve just carried on.*

## Managing their Headaches

While migraines don’t significantly impact their home life, they do adapt differently at work (e.g., in the computer room). Overall, they minimise the impact on their quality of life by using strategies to manage their headaches.

*Res: …like say if I was in the computer room and there was like 29 computers like so if I was sitting next to a student I would have to quickly turn down the brightness on the screen so I can help them with their work on the computer and then I’ll put the brightness back on so they can see it back to normal. So it’s things like that you know it’s because the brightness of the screen was too bright on the screen so they average sort of 20 inch or whatever they are these days and its like that if I was in the workshop and doing DT or wood work and hammering and that like I couldn’t get out of and if I had a migraine it was..I had a migraine and you know it was kind of like just put up with the noise and get on with it you have no choice… mmm… But you just kind of deal with it and adapt to it you just push yourself you know? I’ve got the youngest what 18 months two elder ones which is 9 and 6 so life doesn’t stop… work doesn’t stop… At me new job if I’ve got a migraine I can take a 5 minute sit down or have a cup of tea or have a drink or I can have a few minutes to myself or in the evening ‘cause the other member of staff goes home come 4 c clock there’s only one member of staff in the evening. It’s like being here now, one of them is sitting at the table doing arts and crafts, one’s watching tele, ones’ in his room doing his football stickers or whatever he’s doing… his word search and I can just sit on the sofa and just close my eyes for five you know? I can still be aware of what’s going on or it’s just you have to do it.*

*Int: So was that… were headaches any of the reason for you changing jobs or not?*

*Res: No.*

*Int: No.*

*Res: No I don’t try to use my headaches as an excuse or migraines as an excuse… headaches or migraines I don’t use them as an excuse for everything because there’s a lot of people worse out there than me health wise and they still work and still manage and stuff like that… yeah it does get in the way… I’ve had some like severe one’s where I’ve actually passed out before… that’s not been over… it’s been a fair few years but I’ve learned how to control them and I can manage them I know when one’s coming and stuff like that… mmm… so it’s a bit yeah.*

*Int: So the sort effect of your headaches on work you were saying that you’ve made some adaptations in this job… how’s that been in past jobs?*

*Res: Mmm… well I worked at a school for about 9 years and if I ever had one there it was just kind of like… just ‘crack on’ I’ve got no choice. You know as I said if I was in the computer room at least I could turn down the screen that I was sitting next to the child I was helping at the time and then I started to move to another one there’s like a button on the screen and you just quickly go like that a few times just to dumb down the brightness… mmm… ‘cause a lot of the school environments ……depends what… in most class room environments you can cope with a headache or migraine. If I get a headache or migraines they are totally different.*

*Int: Yeah… yeah that’s really helpful from my point of view. So with work you were saying that there’s certain adaptations or with the other job that you…*

*Res: Yeah well obviously with the classroom it’s like doing English or Humanities or a History lesson or Science there’s not really a massive amount of bangs no little loud noises not really massive bright lights ‘cause it’s like this always under lighting and stuff like that there’s always a certain sort of lighting it’s not too bad ‘cause they don’t use florescent because it’s not good for the students and writing so it’s not too bad it’s manageable… (inaudible) take what I need to take and just get on with it!*

*Int: No ok… so you’ve already told me a bit about this but can you tell me more about how you manage your headaches?*

*Res: Depends on the situation…*

*Int: Ok.*

*Res: … because every situation is always different… mmm… where I am what’s going on it’s always different nothing’s ever the same… mmm… like just then I had to close the blind because of the light was in a certain part of my face… mmm… so yeah.*

*Int: So managing it…*

*Res: Managing it like it depends of where I am like with management if I’m at work… mmm… I can just sit down close for my eyes for a few seconds still sit with them on the sofa and still have a conversation with them but my eyes are just closed and I’m just taking away that brightness and kind of put me head back and just take a nap… steal five minutes you know and it’s life and work still continues because I can still carrying on with my job while doing so… my manager is fully aware of it and she goes yeah fine if I wanna sit there and put me head back for a few minutes closing me eyes take that breather yeah.. Even when I get up it’s still there it’s not gone but I feel that little bit calmer for doing so.*

*Int: Mmm… so how do headaches affect your social life or do they affect your social life?*

*Res: No I don’t let it… but then I’ve had one… I had a migraine… ‘cause me and me mates have just started doing these Escape Rooms…*

*…and when I was doing one I actually had a migraine while I was doing one…*

*…err… it was entertaining… my thought process was a bit slower than usual as I was trying to think and concentrate on stuff so it was hard to think but it didn’t get in the way. Got to push past it. So I’ve learnt to just kind of think you know, life does continue I don’t have any choice. I’m just gonna carry on!*

*Int: Arh… and you were saying sort of the hobbies the hobbies generally does it affect your hobbies you were saying about the gym or…?*

*Res: Mmm… the gym more so yes… mmm… I wouldn’t be able to do the amounts of weights that I would do or running or whatever I have to tone it down a bit.*

*Int: And what’s the thinking behind toning it down?*

*Res: It’s more the pressure I build up because if I am putting a strain on myself like some of the weights you can do like bench pressing some of the weights. I can do on that one like I can do now over a year I would have to take off say about 25 kilograms… 25kg just to bring down the weights so there’s a bigger push and I’m not straining myself to be pushing!*

*Int: So what was your thinking behind that?*

*Res: Because I have had a migraine while being there and I have had excessive weights like I’m trying to push myself like I am… trying to do more so now… to start off with I was just kind of get my body used to it and do everything the basic sort of muscles I not… anyone’s… no-ones used in months. So the past few months I’ve really built it up a bit more and I went there once with a massive migraine… I say migraine and… mmm… try and do the weights and because I was straining to do it… it just hurt even more…*

*Int: Right.*

*Res: …so I just took down the weights… took off… I was actually on the bench press while I was doing that one actually took off 25kg… took off a bit on the inside and then I was able to give it a push but not too much and I didn’t feel it as bad… with the treadmill I wouldn’t do like a good sort of run jog it was more like… err… slowish jog with a bit more of an incline so I’d have to (inaudible) to it…*

*Int: So would there ever be a time where you wouldn’t go or would you always go and do something?*

*Res: I would always go …*

## Medication

Tried Rizatriptan and Zomig in past with no effect. Uses codeine phosphate (Codipar).

*…Yeah because with them tablets when I have them it’s… it depends on the severity of the migraine or headache… sometimes I only need one… it helps to like kinda take the edge off or sometimes I will need the two… then I’ve never gone an actual… consistent time I’ve taken them… sometimes I’ve had a headache or migraine for a few hours and then I would have something… sometimes I’ve had it for a little while and it’s come on really quick and then I’ve took some so it’s never been consistent…*

*…but then I’ll take them then the recommended four hours later take some more if it’s still required but even after that four hours I do sometimes still kind of keep myself going and don’t take it so I don’t want to rely… it can be quite addictive… quite an addictive drug… mmm… because I’ve been on them for a fair few years now the doctors actually stuck them on prescription ‘cause he knows I can manage them and use the drug when I need to. It gets put it on a repeat prescription.*

*…I’m happy with them yeah because sometimes it does take good effect… sometimes they might take a bit longer that’s where obviously the severity of that migraine is more severe hence it obviously… some can last a few hours or some can last a good five or ten hours.*
